# High indoleamine 2,3-dioxygenase transcript levels predict better outcome after front-line cancer immunotherapy

**DOI:** 10.1016/j.isci.2024.109632

**Published:** 2024-03-28

**Authors:** Yu Fujiwara, Shumei Kato, Daisuke Nishizaki, Hirotaka Miyashita, Suzanna Lee, Mary K. Nesline, Jeffrey M. Conroy, Paul DePietro, Sarabjot Pabla, Scott M. Lippman, Razelle Kurzrock

**Affiliations:** 1 Department of Medicine, Roswell Park Comprehensive Cancer Center, Buffalo, NY 14263, USA; 2 Department of Medicine, Icahn School of Medicine at Mount Sinai, Mount Sinai Beth Israel, New York, NY 10003, USA; 3 Center for Personalized Cancer Therapy, University of California, San Diego, Moores Cancer Center, La Jolla, CA 92093, USA; 4 Division of Hematology and Oncology, Dartmouth Cancer Center. One Medical Center Drive, Lebanon, NH 03766, USA; 5 OmniSeq (Labcorp Oncology), Buffalo, NY 14203, USA; 6 MCW Cancer Center and Genomic Sciences and Precision Medicine Center, Medical College of Wisconsin, Milwaukee, WI 53226, USA

**Keywords:** Immunology, Molecular biology, Cancer

## Abstract

Indoleamine 2,3-dioxygenase 1 (IDO1), which catabolizes tryptophan, is a potential target to unlock the immunosuppressive tumor microenvironment. Correlations between IDO1 and immune checkpoint inhibitor (ICI) efficacy remain unclear. Herein, we investigated IDO1 transcript expression across cancers and clinical outcome correlations. High IDO1 transcripts were more frequent in uterine (54.2%) and ovarian cancer (37.2%) but varied between and within malignancies. High IDO1 RNA expression was associated with high expression of PD-L1 (immune checkpoint ligand), CXCL10 (an effector T cell recruitment chemokine), and STAT1 (a component of the JAK-STAT pathway) (all multivariable *p* < 0.05). *PIK3CA* and *CTCF* alterations were more frequent in the high IDO1 group. High IDO1 expression was an independent predictor of progression-free survival (adjusted HR = 0.44, 95% CI 0.20–0.99, *p* = 0.049) and overall survival (adjusted HR = 0.31, 95% CI 0.11–0.87, *p* = 0.026) after front-line ICIs. IDO1 expression warrants further exploration as a predictive biomarker for immunotherapy. Moreover, co-expressed immunoregulatory molecules merit exploration for co-targeting.

## Highlights

• High IDO1 RNA expression is associated with high PD-L1, CXCL10, and STAT1 expression
• *PIK3CA* and *CTCF* alterations occur more often in patients with high IDO1 expression
• High IDO1 mRNA expression is associated with longer survival from immunotherapy
• IDO1 expression merits further exploration as a biomarker for cancer immunotherapy

## Graphical abstract

**Figure:**
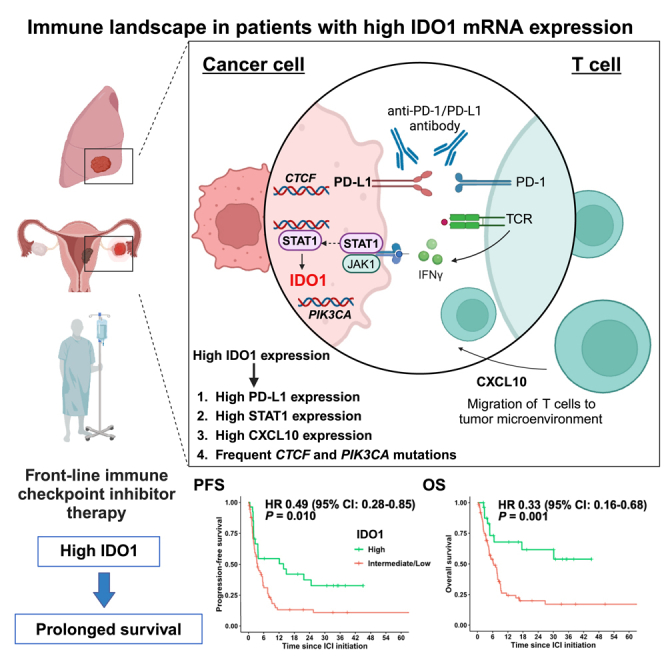

## Introduction

The strategy of blocking inhibitory immune checkpoints such as cytotoxic T lymphocyte-associated protein 4 (CTLA-4), programmed cell death protein 1 (PD-1), programmed death ligand 1 (PD-L1), and lymphocyte activation gene 3 (LAG-3) has revolutionized the landscape of cancer treatment. Agents targeting these checkpoints have been approved for the treatment of patients with advanced cancers, resulting in durable responses, but many patients still develop treatment resistance.^1^

Tryptophan catabolism is recognized as one of the major pathways resulting in an immunosuppressive tumor microenvironment (TME), potentially leading to resistance to immune checkpoint inhibitors (ICIs).^2^ The enzymes including indoleamine 2,3-dioxygenase 1 (IDO1), IDO2, and tryptophan 2,3-dioxygenase catabolize tryptophan to its metabolites such as kynurenine. These metabolites increase myeloid-derived suppressor cells (MDSCs) and regulatory T cells, upregulate PD-1 expression on cytotoxic T cells, and decrease tumor-infiltrating lymphocytes, rendering the TME immunosuppressive.^3^^,^^4^^,^^5^ The production of these enzymes including IDO1 appears to derive primarily from cancer cells and MDSCs, resulting in more immunosuppressive TME.^6^ Additionally, these catabolites serve as a ligand to activate the aryl hydrocarbon receptor (AhR) that induces the accumulation of tumor-associated macrophages, regulatory T cells, and MDSCs, and the conversion of effector T cells to regulatory T cells, resulting in cancer’s ability to evade cellular immunity.^7^^,^^8^^,^^9^ Activation of AhR by tryptophan catabolites is associated with migration and proliferation of cancer cells, whereas knockdown of AhR is associated with an increase in tumor-infiltrating lymphocytes and tumor regression.^10^ Tryptophan can also potentiate cytotoxic T cells against cancer through surface PD-1 downregulation.^11^ Therefore, IDO1 blockade to attenuate tryptophan catabolism has been investigated in order to reverse the immunosuppressive TME, which theoretically could overcome primary resistance to ICI therapy.^8^

Previous research has reported that reduced tryptophan levels are associated with poorer outcomes in cancers such as lung cancer.^12^ Another study (The Cancer Genome Atlas [TCGA] data) showed that high IDO1 expression correlated with shorter survival in patients with colorectal cancer, albeit only in tumors that also demonstrated CD8A expression.^13^ Given these preclinical and clinical data, IDO1 inhibitors/modulators have been developed and evaluated in clinical trials in patients with malignancies. The first-generation IDO1 inhibitor—epacadostat—has been investigated either as monotherapy or in combination with ICIs.^14^ Although epacadostat monotherapy did not show significant efficacy, in combination with the PD-1 inhibitor pembrolizumab, it demonstrated an objective response rate of 40.3% in the overall population (*N* = 26/62) and 61.9% in the malignant melanoma cohort (*N* = 13/21).^15^ These findings supported the initiation of the phase 3 ECHO-301/KEYNOTE-252 trial, which evaluated epacadostat plus pembrolizumab versus pembrolizumab monotherapy in patients with advanced malignant melanoma; unfortunately, this trial did not meet its primary endpoint. Moreover, subgroup analysis based on IDO1 expression did not show an association between IDO1 expression and survival outcomes; however, the threshold for positivity of IDO1 was 1% (defined by immunohistochemistry), which included approximately 90% of the total study population.^16^ In addition to this trial, several other studies have been conducted to evaluate IDO1 inhibitors, but no significant survival benefit has been observed. Thus, there is a need to better understand the landscape of IDO1 in cancers and relationship of IDO expression to immunotherapy outcome. Molecular profiling to elucidate specific mutations and alterations that affect the expression of treatment targets enables delivery of personalized gene-targeted therapies, and immunomic analysis may be of similar interest for immune-targeted treatments.^17^^,^^18^^,^^19^

Our current study aimed to characterize the relationship between IDO1 and other immune molecules as well as outcomes after immune checkpoint blockade in order to better elucidate the biologic and therapeutic implications of IDO1 transcript expression.

## Results

### Patient characteristics

Among 514 patients that were analyzed for immunomic transcript expression, 489 had advanced/metastatic disease and clinically annotated data (Table 1; Tables S1 and S2). Overall, 204 patients (39.7%) were men, median age was 60.8 (interquartile range [IQR]: 50.5–69.5), and the most common tumor types were colorectal cancer (39.7% of 514 patients), pancreatic cancer (10.7%), breast cancer (9.5%), and ovarian cancer (8.4%).

**Table 1:** Association between clinical factors and high (≥75 percentile rank) IDO1 transcriptional expression in 514 patients

Immune markers		*N* of patients	IDO1 group *N* of patients (%)		OR (95% CI), univariate		Adjusted OR (95% CI), multivariate without TMB/MSI (*N* = 514)		Adjusted OR (95% CI), multivariate with MSI without TMB (*N* = 440)		Adjusted OR (95% CI) with TMB (≥10 mutation/mb) included^a^ (*N* = 392)		Adjusted OR (95% CI) with TMB ≥7 (20 mutation/mb) included^a^ (N = 392)		
		514	H (≥75) *N* = 91	I/L (<75) *N* = 423		*p* value		*p* value							p value
Age	≥61	256	56 (21.9)	200 (78.1)	1.78 (1.12–2.84)	0.015	1.04 (0.50–2.14)	0.924	0.90 (0.40–2.03)	0.80	1.31 (0.55–3.10)	0.541	1.31 (0.55–3.07)		0.542
(years)	<60	258	35 (13.6)	223 (86.4)											
Sex	M	204	18 (8.8)	186 (91.2)	0.31 (0.18–0.54)	<0.001	0.51 (0.21–1.24)	0.137	0.41 (0.15–1.15)	0.091	0.38 (0.11–1.27)	0.116	0.38 (0.11,1.28)		0.118
	F	310	73 (23.5)	237 (76.5)											
CD38	H	79	35 (44.3)	44 (55.7)	5.38 (3.18–9.10)	<0.001	0.79 (0.24–2.62)	0.695	1.22 (0.30–4.93)	0.778	0.61 (0.14–2.70)	0.517	0.54 (0.12–2.38)		0.412
	I/L	435	56 (12.9)	379 (87.1)											
CD39	H	131	29 (22.1)	102 (77.9)	1.47 (0.90–2.41)	0.144									
	I/L	383	62 (16.2)	321 (83.8)											
VEGF-A	H	134	19 (14.2)	115 (85.8)	0.71 (0.41–1.22)	0.238									
	I/L	380	72 (18.9)	308 (81.1)											
AXL	H	144	24 (16.7)	120 (83.3)	0.90 (0.54–1.51)	0.797									
	I/L	370	67 (18.1)	303 (81.9)											
TGFB1	H	130	28 (21.5)	102 (78.5)	1.40 (0.85–2.30)	0.186									
	I/L	384	63 (16.4)	321 (83.6)											
CD80	H	115	34 (29.6)	81 (70.4)	2.52 (1.54–4.11)	<0.001	0.55 (0.19–1.59)	0.272	0.58 (0.18–1.88)	0.367	0.22 (0.05–1.06)	0.059	0.25 (0.05–1.19)		0.081
	I/L	399	57 (14.3)	342 (85.7)											
CD86	H	84	30 (35.7)	54 (64.3)	3.36 (1.99–5.66)	<0.001	2.33 (0.55–9.84)	0.249	1.64 (0.30–8.85)	0.567	1.88 (0.29–12.16)	0.509	1.65 (0.26–10.68)		0.597
	I/L	430	61 (14.2)	369 (85.8)											
CTLA-4	H	87	38 (43.7)	49 (56.3)	5.47 (3.28–9.13)	<0.001	1.39 (0.41–4.73)	0.60	1.19 (0.31–4.58)	0.80	3.52 (0.72–17.32)	0.121	3.7 (0.76–18.11)		0.106
	I/L	427	53 (12.4)	374 (87.6)											
LAG-3	H	116	54 (46.6)	62 (53.4)	8.50 (5.17–13.98)	<0.001	1.40 (0.58–3.37)	0.45	1.19 (0.44–3.23)	0.729	1.56 (0.56–4.40)	0.397	1.48 (0.52–4.16)		0.462
	I/L	398	37 (9.3)	361 (90.7)											
PD-1	H	93	43 (46.2)	50 (53.8)	6.68 (4.03–11.09)	<0.001	1.43 (0.46–4.50)	0.538	1.68 (0.47–5.98)	0.424	2.03 (0.49–8.31)	0.326	2.13 (0.52–8.74)		0.296
	I/L	421	48 (11.4)	373 (88.6)											
PD-L1	H	67	39 (58.2)	28 (41.8)	10.58 (6.01–18.62)	<0.001	4.09 (1.38–12.11)	**0.011**	5.27 (1.55–17.87)	**0.008**	4.80 (1.25–18.37)	**0.022**	4.80 (1.24–18.58)		**0.023**
	I/L	447	52 (11.6)	395 (88.4)			**High PD-L1 expression was significantly associated with high IDO1 expression**								
PD-L2	H	100	37 (37.0)	63 (63.0)	3.92 (2.38–6.43)	<0.001	1.01 (0.35–2.90)	0.985	0.64 (0.18–2.26)	0.486	0.88 (0.23–3.42)	0.859	0.91 (0.24–3.49)	0.887	
	I/L	414	54 (13.0)	360 (87.0)											
TIGIT	H	99	45 (45.5)	54 (54.5)	6.68 (4.05–11.03)	<0.001	0.68 (0.17–2.63)	0.575	0.46 (0.10–2.17)	0.328	1.34 (0.25–7.11)	0.731	1.44 (0.27–7.74)	0.670	
	I/L	415	46 (11.1)	369 (88.9)											
TIM3	H	90	26 (28.9)	64 (71.1)	2.24 (1.32–3.80)	0.004	0.45 (0.11–1.88)	0.271	0.62 (0.11–3.38)	0.581	0.73 (0.14–3.94)	0.718	0.71 (0.13–3.88)	0.696	
	I/L	424	65 (15.3)	359 (84.7)											
VISTA	H	166	31 (18.7)	135 (81.3)	1.10 (0.68–1.78)	0.712									
	I/L	348	60 (17.2)	288 (82.8)											
IDO2	H	83	20 (24.1)	63 (75.9)	1.61 (0.92–2.83)	0.115									
	I/L	431	71 (16.5)	360 (83.5)											
TDO2	H	159	29 (18.2)	130 (81.8)	1.05 (0.65–1.72)	0.901									
	I/L	355	62 (17.5)	293 (82.5)											
IFNγ	H	51	35 (68.6)	16 (31.4)	15.7 (7.91–32.6)	<0.001	2.72 (0.76–9.78)	0.126	2.82 (0.60–13.18)	0.187	3.52 (0.72–17.23)	0.121	3.42 (0.69–16.8)	0.131	
	I/L	463	56 (12.1)	407 (87.9)											
IL-6	H	121	19 (15.7)	102 (84.3)	0.83 (0.48–1.44)	0.587									
	I/L	393	72 (18.3)	321 (81.7)											
STAT1	H	103	59 (57.3)	44 (42.7)	15.88 (9.33–27.02)	<0.001	4.08 (1.76–9.46)	**0.001**	4.64 (1.78–12.09)	**0.002**	7.12 (2.37–21.38)	**<0.001**	6.94 (2.36–20.42)	**<0.001**	
	I/L	411	32 (7.8)	379 (92.2)			**High STAT1 expression was significantly associated with high IDO1 expression**								
STAT3	H	111	24 (21.6)	87 (78.4)	1.38 (0.82–2.33)	0.261									
	I/L	403	67 (16.6)	336 (83.3)											
CCR1	H	96	26 (27.1)	70 (72.9)	2.02 (1.20–3.40)	0.011	0.53 (0.16–1.78)	0.303	0.60 (0.16–2.32)	0.462	0.29 (0.05–1.66)	0.164	0.30 (0.05–1.73)	0.179	
	I/L	418	65 (15.6)	353 (84.4)											
CCR2	H	119	37 (31.1)	82 (68.9)	2.85 (1.76–4.62)	<0.001	0.42 (0.13–1.32)	0.137	0.23 (0.06–0.91)	**0.036**	0.36 (0.09–1.45)	0.152	0.33 (0.08–1.34)	0.122	
	I/L	395	54 (13.7)	341 (86.3)											
CXCR2	H	110	20 (18.2)	90 (81.8)	1.04 (0.60–1.80)	0.888									
	I/L	404	71 (17.6)	333 (82.4)											
AKT1	H	176	31 (17.6)	145 (82.3)	0.99 (0.61–1.60)	1									
	I/L	338	60 (17.8)	278 (82.2)											
MTOR	H	130	25 (19.2)	105 (80.8)	1.15 (0.69–1.91)	0.597									
	I/L	384	66 (17.2)	318 (82.8)											
PIK3CA	H	138	28 (20.3)	110 (79.7)	1.26 (0.77–2.08)	0.363									
	I/L	376	63 (16.8)	313 (83.2)											
CD28	H	102	25 (24.5)	77 (75.5)	1.70 (1.01–2.87)	0.059									
	I/L	412	66 (16.0)	346 (84.0)											
CD40	H	114	40 (35.1)	74 (64.9)	3.70 (2.28–6.00)	<0.001	2.36 (1.05–5.31)	**0.038**	2.61 (1.06–6.45)	**0.037**	2.45 (0.89–6.73)	0.083	2.45 (0.91–6.61)	0.076	
	I/L	400	51 (12.8)	349 (87.3)											
GITR	H	99	34 (34.3)	65 (65.7)	3.29 (1.99–5.42)	<0.001	1.25 (0.49–3.20)	0.646	1.21 (0.41–3.62)	0.730	1.16 (0.36–3.73)	0.804	1.16 (0.37–3.68)	0.799	
	I/L	415	57 (13.7)	358 (86.3)											
ICOS	H	70	34 (48.6)	36 (51.4)	6.41 (3.72–11.06)	<0.001	1.64 (0.46–5.78)	0.443	1.67 (0.40–3.62)	0.486	1.02 (0.19,5.48)	0.984	1.11 (0.21–5.82)	0.903	
	I/L	444	57 (12.8)	387 (87.2)											
ICOSLG	H	192	31 (16.1)	161 (83.9)	0.84 (0.52–1.35)	0.551									
	I/L	322	60 (18.6)	262 (81.4)											
OX40	H	122	31 (25.4)	91 (74.6)	1.88 (1.15–3.08)	0.014	0.60 (0.22–1.62)	0.310	0.52 (0.18–1.56)	0.245	0.49 (0.15,1.61)	0.242	0.48 (0.15–1.60)	0.23	
	I/L	392	60 (15.3)	332 (84.7)											
OX40L	H	119	27 (22.7)	92 (77.3)	1.52 (0.92–2.52)	0.131									
	I/L	395	64 (16.2)	331 (83.8)											
CSF1R	H	115	25 (21.7)	90 (78.3)	1.40 (0.84–2.35)	0.213									
	I/L	399	66 (16.5)	333 (83.5)											
CXCR4	H	110	27 (24.5)	83 (75.5)	1.73 (1.04–2.88)	0.048	0.63 (0.24–1.68)	0.354	0.89 (0.31–2.51)	0.820	0.57 (0.17,1.85)	0.346	0.58 (0.18–1.87)	0.336	
	I/L	404	64 (15.8)	340 (84.2)											
CD8	H	89	42 (47.2)	47 (52.8)	6.86 (4.11–11.44)	<0.001	1.34 (0.40–4.47)	0.634	1.03 (0.26–4.04)	0.969	0.99 (0.23–4.28)	0.991	0.95 (0.22–4.13)	0.940	
	I/L	425	49 (11.5)	376 (88.5)											
CXCL9	H	71	47 (66.2)	24 (33.8)	17.76 (9.92–31.78)	<0.001	1.86 (0.58–5.92)	0.293	2.15 (0.57–8.04)	0.256	0.82 (0.18–3.81)	0.804	0.85 (0.19–3.89)	0.833	
	I/L	443	44 (9.9)	399 (90.1)											
CXCL10	H	96	56 (58.3)	40 (41.7)	15.32 (8.99–26.11)	<0.001	4.91 (1.91–12.62)	**<0.001**	6.55 (2.00–26.19)	**<0.001**	4.63 (1.43–14.95)	**0.010**	4.97 (1.54–16.02)	**0.007**	
	I/L	418	35 (8.4)	383 (91.6)			**High CXCL10 expression was significantly associated with high IDO1 expression**								
CCR4	H	131	37 (28.2)	94 (71.8)	2.40 (1.49–3.86)	<0.001	2.13 (0.81–5.57)	0.123	2.05 (0.68–6.23)	0.210	3.17 (0.9,11.24)	0.074	3.26 (0.91–11.67)	0.07	
	I/L	383	54 (14.1)	329 (85.9)											
CCR5	H	96	34 (35.4)	62 (64.6)	3.47 (2.10–5.74)	<0.001	0.59 (0.17–2.05)	0.404	0.82 (0.20–3.41)	0.783	1.15 (0.23–5.67)	0.866	1.28 (0.26–6.35)	0.766	
	I/L	418	57 (13.6)	361 (86.4)											
FOXP3	H	123	47 (38.2)	76 (61.8)	4.88 (3.02–7.88)	<0.001	2.77 (1.01–7.63)	**0.048**	3.62 (1.17–11.15)	**0.025**	2.73 (0.73–10.16)	0.134	2.53 (0.68,9.42)	0.167	
	I/L	391	44 (11.3)	347 (88.7)											
MSI (N = 440)	H	15	8 (53.3)	7 (46.7)	5.08 (1.79–14.44)	0.003			6.8 (0.88–52.50)	0.066					
	L	425	78 (18.4)	347 (81.6)											
TMB (N = 392)	H	48	17 (35.4)	31 (64.6)	3.01 (1.56–5.83)	0.002					1.72 (0.46–6.35)	0.417			
Cutoff: <10 mutations/mb	L	344	53 (15.4)	291 (84.6)											
TMB (N = 392)	H	22	8 (36.4)	14 (63.6)	2.84 (1.14–7.06)	0.038							3.87 (0.70–21.43)	0.121	
Cutoff: <20 mutations/mb	L	370	62 (16.8)	308 (83.2)											
Bladder	Yes	4	0 (0.0)	4 (100.0)	0 (0.00-Inf)	1									
	No	510	91 (17.8)	419 (82.2)											
Breast	Yes	49	8 (16.3)	41 (83.7)	0.90 (0.41–1.99)	1									
	No	465	83 (17.8)	382 (82.2)											
Colorectal	Yes	140	19 (13.6)	121 (86.4)	0.66 (0.38–1.14)	0.154									
	No	374	72 (19.3)	302 (80.7)											
CUP	Yes	13	2 (15.4)	11 (84.6)	0.84 (0.18–3.86)	1									
	No	501	89 (17.8)	412 (82.2)											
Esophageal	Yes	17	3 (17.6)	14 (82.4)	1.00 (0.28–3.54)	1									
	No	497	88 (17.7)	409 (82.3)											
Gastric	Yes	25	3 (12.0)	22 (88.0)	0.62 (0.18–2.12)	0.595									
	No	489	88 (18.0)	401 (82.0)											
Hepatobiliary	Yes	19	0 (0.0)	19 (100.0)	0 (0.00-Inf)	0.033	0.00 (0.00-Inf)	0.985	0 (0-Inf)	0.986	0 (0-Inf)	0.987	0 (0-Inf)	0.987	
	No	495	91 (18.4)	404 (81.6)											
HNSCC	Yes	12	1 (8.3)	11 (91.7)	0.42 (0.05–3.26)	0.702									
	No	502	90 (17.9)	412 (82.1)											
Lung	Yes	20	5 (25.0)	15 (75.0)	1.58 (0.56–4.47)	0.373									
	No	494	86 (17.4)	408 (82.6)											
Melanoma	Yes	7	2 (28.6)	5 (71.4)	1.88 (0.36–9.84)	0.36									
	No	507	89 (17.6)	418 (82.4)											
Neuroendocrine	Yes	15	2 (13.3)	13 (86.7)	0.71 (0.16–3.20)	1									
	No	499	89 (17.8)	410 (82.2)											
Ovarian	Yes	43	16 (37.2)	27 (62.8)	3.13 (1.61–6.09)	0.001	3.94 (1.34–11.55)	**0.012**	4.85 (1.41–16.46)	**0.011**	5.04 (1.43–17.71)	**0.012**	5.90 (1.64–21.22)	**0.007**	
	No	471	75 (15.9)	396 (84.1)			**Ovarian cancer was associated with high IDO1 expression**								
Pancreatic	Yes	55	5 (9.1)	50 (90.9)	0.43 (0.17–1.12)	0.092									
	No	459	86 (18.7)	373 (81.3)											
Prostate	Yes	4	1 (25.0)	3 (75.0)	1.56 (0.16–15.13)	0.542									
	No	510	90 (17.6)	420 (82.4)											
RCC	Yes	3	1 (33.3)	2 (66.7)	2.34 (0.21–26.07)	0.443									
	No	511	90 (17.6)	421 (82.4)											
Sarcoma	Yes	24	3 (12.5)	21 (87.5)	0.65 (0.19–2.24)	0.783									
	No	490	88 (18.0)	402 (82.0)											
Small intestine	Yes	12	1 (8.3)	11 (91.7)	0.42 (0.05–3.26)	0.702									
	No	502	90 (17.9)	412 (82.1)											
Uterine	Yes	24	13 (54.2)	11 (45.8)	6.24 (2.70–14.44)	<0.001	18.51 (5.43–63.11)	**<0.001**	25.67 (6.6–99.84)	**<0.001**	25.77 (5.62–118.16)	**<0.001**	29.21 (6.18–137.97)	**<0.001**	
	No	490	78 (15.9)	412 (84.1)			**Uterine cancer was associated with high IDO1 expression**								

Univariate and multivariate analysis of odds ratio (OR) for high IDO1 expression^a^.

The RNA expression of the selected immune factors was calculated and the transcript abundance of these molecules was normalized and compared to the internal reference consisting of 735 tumors spanning 35 histologies. Rank values of each selected factor were determined on a scale of 1–100 as previously reported. Rank values were categorized as low (0–24), intermediate (25–74), and high (75–100).

*p* values ≤0.05 in univariate analysis were selected for multivariate analysis.

Factors with *p* values of ≤ 0.05 in multivariable analysis are shown in bold.

AKT1, Ak strain transforming 1; CCR, C-C motif chemokine receptor; CD, clusters of differentiation; CI, confidence interval; CTLA-4, cytotoxic T lymphocyte-associated antigen 4; CSF1R, macrophage colony stimulating factor 1 receptor; CUP, cancer of unknown primary; CXCL, CXC chemokine ligand; CXCR, CXC chemokine receptors; FOXP3, forkhead box protein 3; GITR, glucocorticoid-induced TNF receptor family-related protein; H, high; HNSCC, head and neck squamous cell carcinoma; ICOS, inducible T cell costimulator; ICOSLG, ICOS ligand; IDO, indolamine-2,3-dioxygenase; IFNγ, interferon gamma; I/L, intermediate/low; IL-6, interleukin-6; LAG-3, lymphocyte activation gene 3; MSI, microsatellite instability; MTOR, mammalian target of rapamycin; OR, odds ratio; OX40L, OX40 ligand; PIK3CA, phosphatidylinositol-4,5-bisphosphate 3-kinase catalytic subunit alpha; RCC, renal cell carcinoma; PD-1, programmed cell death protein 1; PD-L1, programmed death-ligand 1; PD-L2, programmed death-ligand 2; STAT, signal transducer and activator of transcription; TDO2, tryptophan 2,3-dioxygenase; TGFB1, Transforming growth factor beta; TIGIT, T cell immunoglobulin and ITIM domain; TIM3, T cell immunoglobulin domain and mucin domain 3; TMB, tumor mutational burden; VEGF-A, vascular endothelial growth factor A; VISTA, V-domain Ig suppressor of T cell activation.

a 514 patients were analyzed in the multivariate analysis; a second multivariate analysis was performed by excluding MSI and TMB information (*N* = 514); a third analysis contained MSI but not TMB information (*N* = 440); fourth and fifth analyses included TMB (≥10 mutations/mb, (≥10 mutations/mb as a cutoff to define high and low, respectively) but not MSI information (*N* = 392).

Altogether, 217 patients were treated with ICIs, mainly with an anti-PD-1 inhibitor or anti-PD-L1 inhibitor; 102 patients received first-line ICI treatment and 115 were treated in subsequent line (second line and beyond). Among the 102 patients who received first-line ICI, 46 (45.1%) were men, the median age was 61.9 years (IQR: 54.4–70.8), and the most common tumors were colorectal (5.4% of 514 patients), pancreatic (1.9%), breast (1.6%), and hepatobiliary cancer (1.4%).

### IDO1 expression varied between and within tumor types, with uterine and ovarian cancers having the highest proportion of high IDO1 expressors

**Figure 1:**
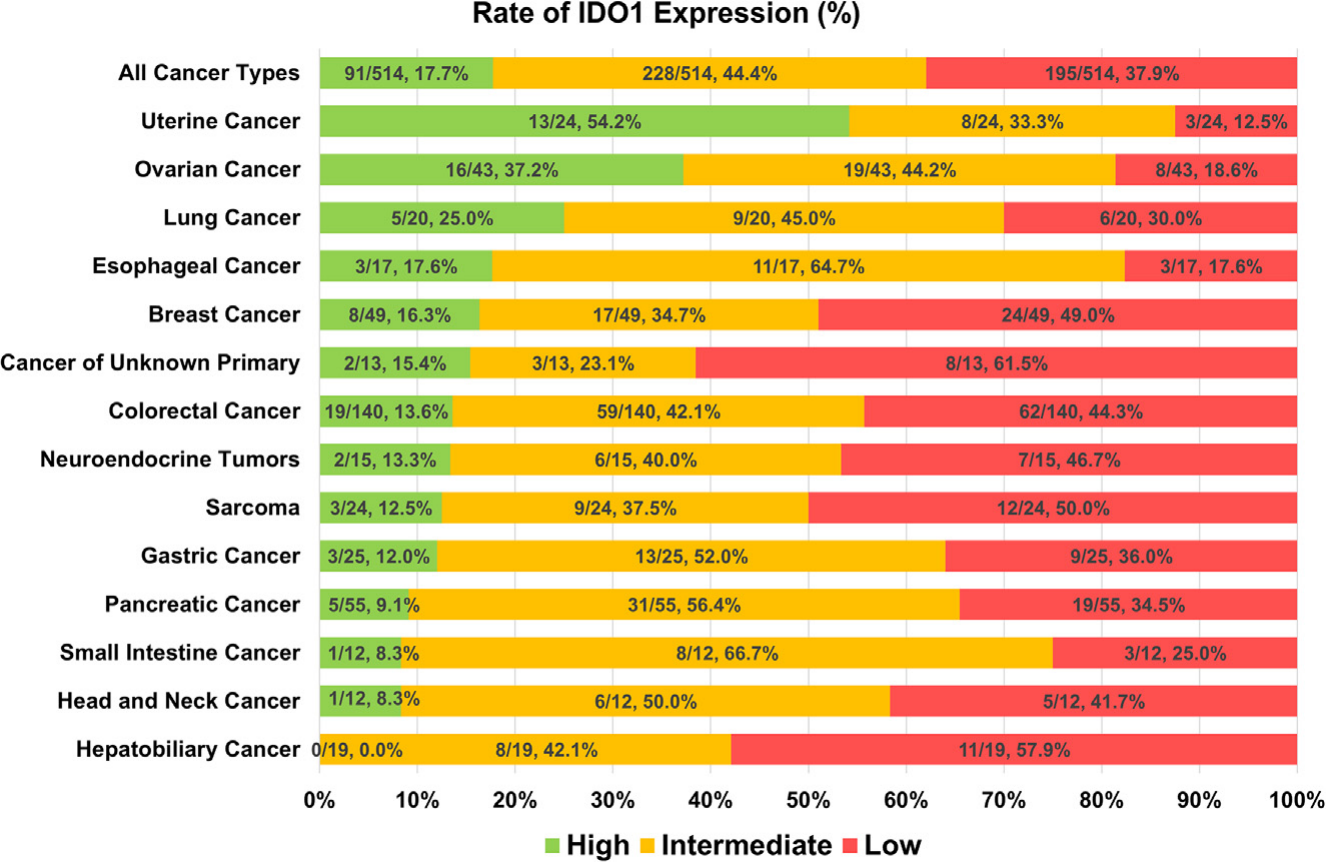
IDO1 RNA expression according to the cancer type Cancer types containing 10 or more patients are shown. Total number of patients is 514. Next-generation sequencing was applied to assess IDO1 RNA expression using 735 tumors spanning 35 histologies as a reference, and then rank values were determined on a 1 to 100 scale. Rank values were categorized into low (green: 0–24), intermediate (yellow: 25–74), and high (red: 75–100]). The percentage of patients with each IDO1 classification level (high, intermediate, and low) is shown in this graph. Uterine cancer had the highest proportion of tumors that expressed high IDO1 levels, followed by ovarian and lung cancer. Lung cancer type (Number of patients: High IDO1/Overall): Adenocarcinoma (4/13), small-cell lung cancer (1/2), non-small-cell lung cancer not otherwise specified (1/1), Squamous cell carcinoma (0/2), Sarcomatoid carcinoma (0/1), Mesothelioma (0/1). IDO1, Indoleamine 2,3-dioxygenase 1; RNA, Ribonucleic acid.

To evaluate IDO1 expression, we first performed transcriptomic expression evaluation on 514 metastatic/advanced tumors at the University of California San Diego Moores Cancer Center. The percentile of IDO1 RNA expression from each tumor was determined as compared to 735 control tumors spanning 35 histologies, and each value was classified by percentile rank as low (0–24), intermediate (25–74), and high (75–100). In the entire cohort of 514 patients, IDO1 expression of 91 patients (17.7%) was classified as high, 228 (44.4%) as intermediate, and 195 (37.9%) as low. Regarding tumor type, high IDO1 expression was commonly seen in uterine cancer (54.2% of uterine tumors), followed by ovarian (37.2%), lung (25.0%), esophageal (17.6%), and breast cancer (16.3%) (Figure 1). Still, there was considerable variability in IDO1 expression within cancer types with, for instance, 12.5% of uterine cancers expressing low IDO1. Hepatobiliary cancers had no high expressors, albeit with only 19 tumors tested.

### High IDO1 expression often co-segregated with high expression of immune checkpoints across cancers

**Figure 2:**
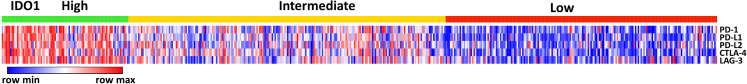
A heatmap illustrating expression percentiles of selected immune factors based on IDO1 high/intermediate/low expression Immune checkpoints (PD-1, PD-L1, PD-L2, CTLA-4, and LAG-3) are mapped according to IDO1 expression. The heatmap shows relatively higher expression of immune checkpoints in the IDO1 high group, heterogeneous or mixture of high and low expression in the IDO1 intermediate group, and relatively low expression in the IDO1 low group. CTLA-4, cytotoxic T lymphocyte-associated antigen 4; IDO1, indoleamine 2,3- dioxygenase 1; LAG-3, lymphocyte activation gene 3; PD-1, programmed cell death protein 1; PD-L1, programmed death-ligand 1; PD-L2, programmed death-ligand 2.

Next, immune markers related to the tumor immune microenvironment were selected (Resource S1), and the association between their RNA expression and IDO1 RNA expression was analyzed. Immune checkpoints targeted by the Food and Drug Administration-approved ICIs (PD-1, PD-L1, PD-L2, CTLA-4, and LAG-3) were first assessed according to IDO1 expression groups (high, intermediate, and low). The heatmap shows higher expression of these checkpoints in the high IDO1 group, heterogeneous expression in the intermediate IDO1 group, and relatively low expression in the low IDO1 group (Figure 2), suggesting a close relationship between IDO1 and immune checkpoints.

### High IDO1 expression correlated independently and significantly with expression of high PD-L1, STAT1, and CXCL10 transcripts and correlation was linear per TCGA dataset

To further investigate associations between IDO1 and immune factors, odds ratios for high IDO1 expression based on selected immune markers (high vs. intermediate/low), microsatellite instability (high vs. stable), tumor mutational burden (TMB) (high vs. low, using 10 or 20 mutations/mb as cutoff), and cancer types were calculated and compared using univariate and multivariate analyses (Table 1).

In univariate analysis, high expression of several immune markers such as those related to inhibitory immune checkpoints, co-stimulatory immune checkpoints, MDSCs, and regulatory T cells were associated with high IDO1 expression. Multivariable analysis showed an independent association between high IDO1 and high PD-L1, high CXCL10, high STAT1, and between high IDO1 and ovarian and uterine cancers; tumors in men were significantly associated with lower IDO1 levels (all multivariable *p* values <0.05, Table 1).

Correlation between IDO1 versus CXCL10 or versus STAT1 or versus PD-L1 was also compared by using TCGA data from 1,210 samples that included mRNA expression information to validate findings from our dataset. Correlation was tested using Spearman test with a *p* value threshold of 0.05 for significance. CXCL10 (Spearman R = 0.70, p < 0.001), STAT1 (Spearman R = 0.53, p < 0.001), and PD-L1 (Spearman R = 0.44, p < 0.001) were all correlated with IDO1 expression, confirming that there is a monotonic association between these variables (Figure S1) in addition to the dichotomous association (high vs. medium/low) demonstrated by our dataset. Among these, CXCL10 showed the strongest correlation with IDO1 expression.

### Genomic alterations in *PIK3CA* and *CTCF* genes were more frequent in the high IDO1 expressing tumors

The frequency of genomic alterations was compared between high and intermediate/low IDO1 expression groups to characterize the landscape of gene alteration. In the high IDO1 group, alterations of *TP53* (53%), *ARID1A* (23%), *PIK3CA* (23%), *KRAS* (20%), and *PTEN* (15%) were frequently observed. In the intermediate/low IDO1 group, *TP53* (55%), *KRAS* (26%), *APC* (21%), *SMAD4* (11%), *and ARID1A* (10%) were commonly altered.

**Figure 3:**
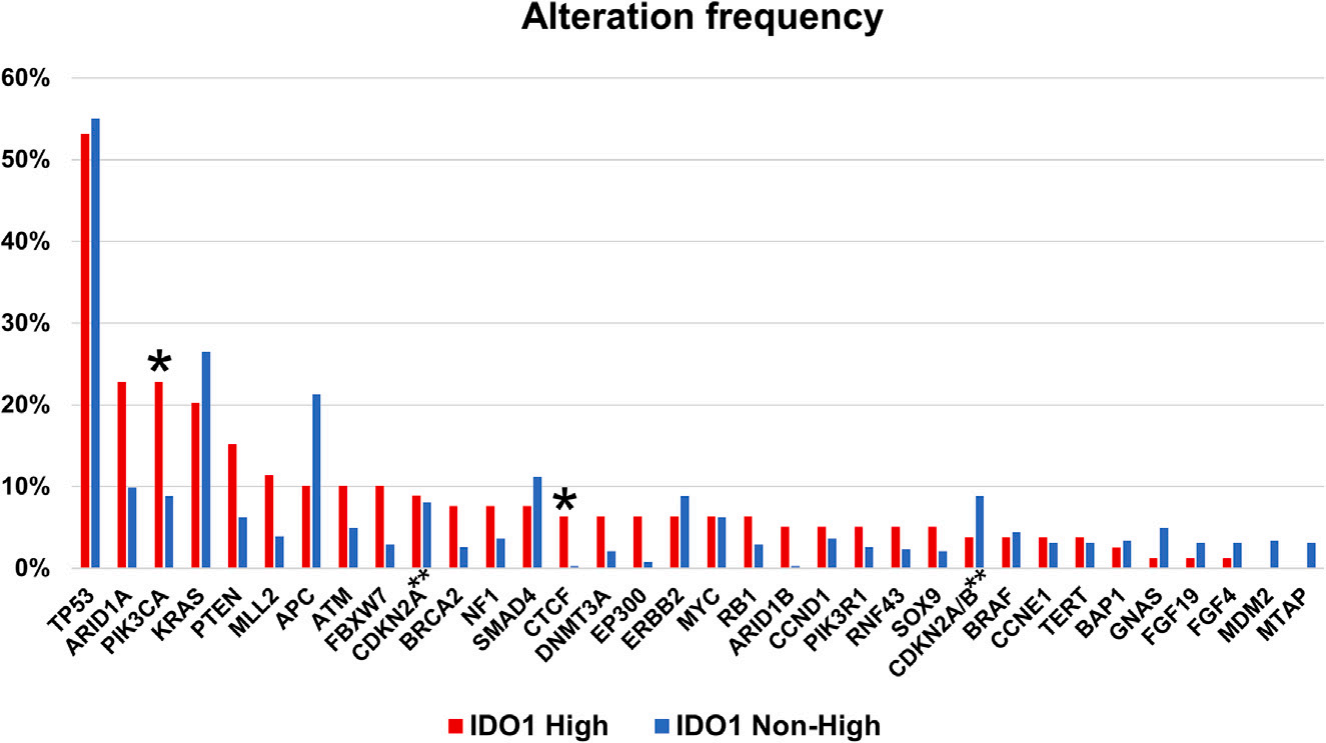
Genomic alteration frequency according to IDO1 high (≥75^th^ RNA percentile rank*)* and intermediate/low (<75th percentile RNA rank) expression Frequency of gene alterations was calculated in groups with high and intermediate/low (non-high) IDO1 RNA expression. Among 514 patients, gene alteration information was available in 79 patients with high IDO1 expression and 385 patients with non-high IDO1 expression. Frequency of each gene alteration was compared using the Fisher’s exact test with a correction with Bonferroni method for multiple comparison; *p* < 0.001471 (=0.05/34) was considered statistically different. The graph includes the top 20 gene alteration frequency from each IDO1 high and non-high group and contains a total of 34 genes to be compared between high and non-high IDO1 expression groups. ∗: Frequency was statistically higher in the high IDO1 group. After Bonferroni correction for multiple comparison, PIK3CA and CTCF alterations were associated with high IDO1 RNA expression. ∗∗: Mutations only in CDKN2A were categorized into CDKN2A. CDKN2A/B homozygous deletion was shown as CDKN2A/B separately. Abbreviations: IDO1, indoleamine 2,3-dioxygenase 1.

When comparing alteration frequency between these two groups, alterations in *PIK3CA* and *CTCF* (a transcriptional regulator) were significantly more frequent in the high versus non-high IDO1 group (after Bonferroni adjustment for multiple comparisons) (Figure 3). As a correlation between *PIK3CA* and *IDO1* alterations was observed, the transcriptomic expression of AKT1, MTOR, and PIK3CA was assessed based on IDO1 expression. In our entire cohort (*n* = 514), none of these factors in the PI3K pathway were significantly associated with the high IDO1 expression group (Table 1). The correlation between IDO1 versus AKT1, MTOR, and PIK3CA was also evaluated from the TCGA data. MTOR was slightly correlated (Spearman R = −0.15, *p* < 0.001), but AKT1 (Spearman R = 0.03, *p* = 0.312) and PIK3CA (Spearman R = −0.04, *p* = 0.183) were not dichotomously correlated with IDO1 mRNA expression (Figure S2).

### IDO1 expression was not a prognostic factor for OS from the time of advanced/metastatic disease in immunotherapy-naive patients

**Figure 4:**
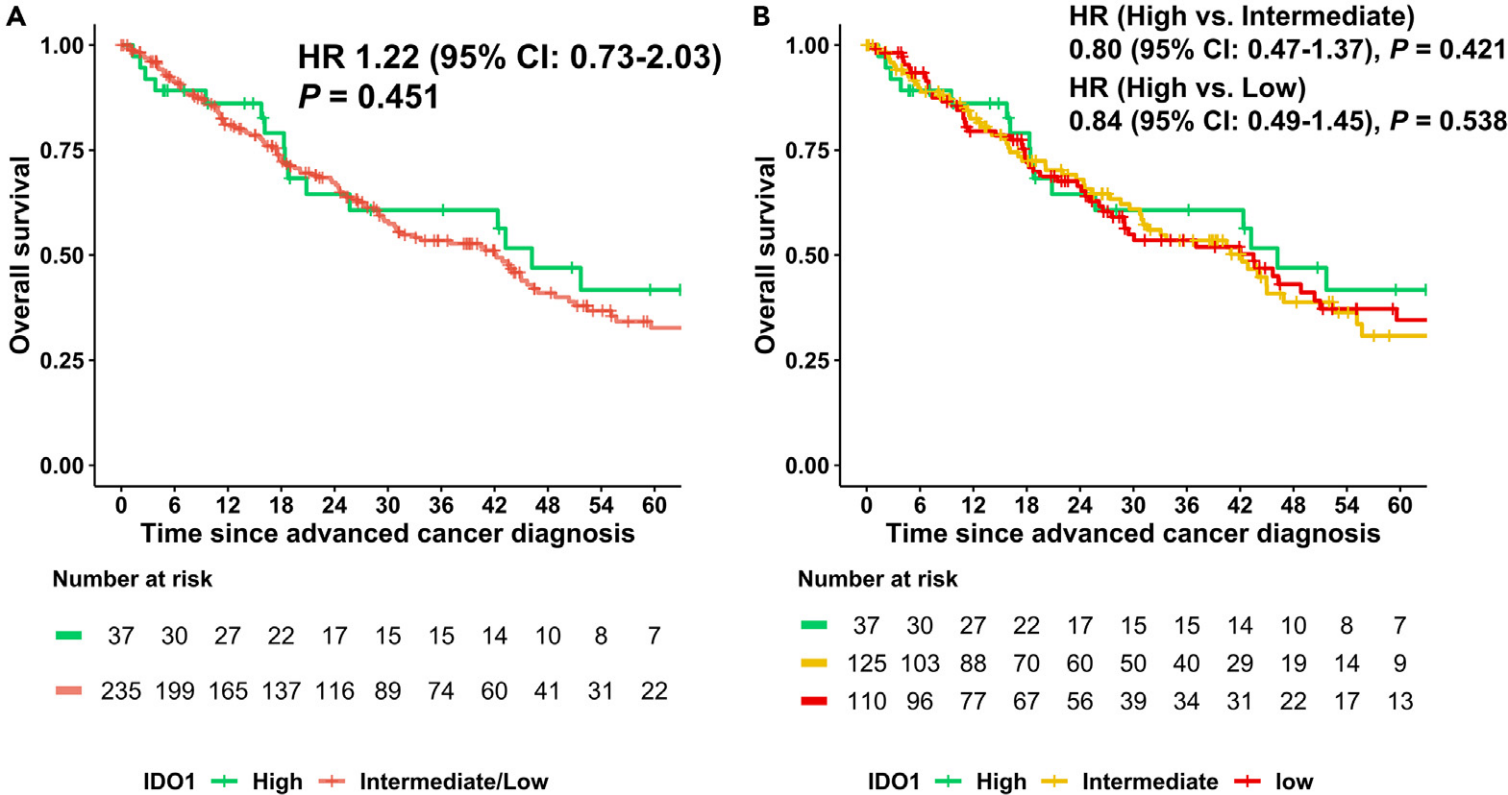
Overall survival from the time of advanced cancer diagnosis based on IDO1 expression in advanced solid tumors not treated with immune checkpoint inhibitors (*n* = 272) Overall survival based on IDO1 expression in patients with advanced solid tumors not treated with immune checkpoint inhibitors (*n* = 272). The log-rank test was used to assess differences in Kaplan-Meier curves. P value < 0.05 was considered statistically different. X axis: Time since advanced cancer diagnosis. Y axis: Overall survival. Expression profiles were stratified by rank values into “low” (0–24), “intermediate” (25–74), and “high” (75–100). (A) The Kaplan Meier curve according to IDO1 high (*n* = 37) and intermediate/low (n = 235) group. HR = 1.22 (95% CI: 0.73–2.03, *p* = 0.451). (B) The Kaplan Meier curve according to IDO1 high (*n* = 37), intermediate (*n* = 125), low (*n* = 110) group. p = 0.723 for entire groups. HR = 0.80 (high vs. intermediate, 95% CI: 0.47–1.37, *p* = 0.421), 0.84 (high vs. low, 95% CI: 0.49–1.45, *p* = 0.538). This figure shows that IDO1 levels did not correlate with prognosis from the time of metastatic disease. 95% CI, 95% confidence interval; HR, hazard ratio; IDO1, Indoleamine 2,3- dioxygenase 1.

In the 272 pan-cancer patients who never received immunotherapy, overall survival (OS) from time of advanced/metastatic disease was not correlated with IDO1 level. The Kaplan-Meier curve according to IDO1 high (*n* = 37) vs. intermediate/low (*n* = 235) group showed a hazard ratio (HR) = 1.22 (95% confidence interval [CI]: 0.73–2.03, *p* = 0.451) (Figure 4, panel A); also, when divided into IDO1 high, intermediate, and low groups, no differences in OS were seen (Figure 4, panel B).

The largest set of patients in our database had advanced/metastatic colorectal cancer. High IDO1 was not associated with OS in the entire cohort and in the immunotherapy-naive colorectal population as follows: OS from advanced/metastatic disease regardless of immunotherapy treatment (IDO high: *n* = 17, IDO intermediate/low: *n* = 116), HR = 0.94 (95% CI: 0.47–1.90, *p* = 0.87) and OS from advanced/metastatic disease in immunotherapy-naive patients (IDO high: *n* = 11, IDO intermediate/low: *n* = 57), HR = 1.43 (95% CI: 0.62–3.29, *p* = 0.4).

### High IDO1 expression predicts longer PFS and OS after first-line, but not later-line, immune checkpoint inhibitor therapy

**Figure 5:**
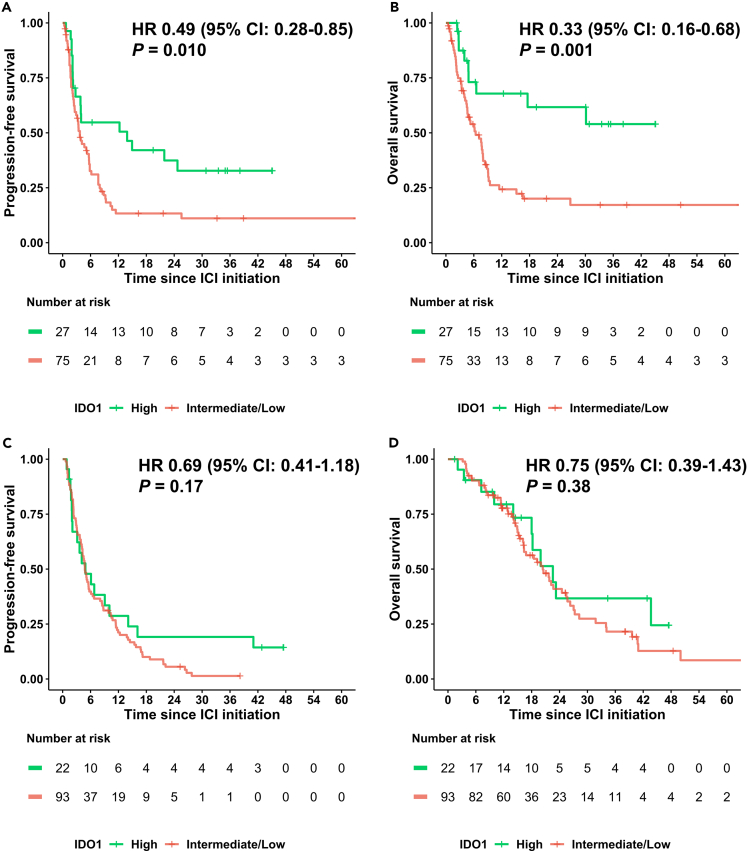
Progression-free survival and overall survival based on IDO1 expression in patients treated with immune checkpoint inhibitors in the first-line and second-line/beyond setting (A and B) PFS and OS in patients treated with immune checkpoint inhibitors in the first-line setting (*n* = 102 patients). (C and D) PFS and OS in patients treated with immune checkpoint inhibitors in the second-line/beyond setting (*n* = 115). The figure shows significantly longer PFS and OS in the IDO1 high RNA expression group vs. low/intermediate expression group after first-line but not later-line immune checkpoint inhibitor therapy. The log-rank test was used to assess differences in Kaplan-Meier curves. P value < 0.05 was considered statistically different. X axis: Time since initiation of immune checkpoint blockade. Y axis: PFS (A, C), OS (B, D). Expression profiles were stratified by rank values into “low” (0–24), “intermediate” (25–74), and “high” (75–100). Panels A and B: The Kaplan Meier curves according to IDO1 high (*n* = 27) and intermediate/low (*n* = 75) group. (A) PFS HR = 0.49 (95% CI: 0.28–0.85, *p* = 0.010). (B) OS HR = 0.33 (95% CI: 0.16–0.68, *p* = 0.001). (C and D) The Kaplan Meier curves according to IDO1 high (*n* = 27) and intermediate/low (*n* = 75) group. (C) PFS HR = 0.69 (95% CI: 0.41–1.18, *p* = 0.175). (D) OS HR = 0.75 (95% CI: 0.39–1.43, p = 0.382). 95% CI, 95% confidence interval; HR, hazard ratio; IDO1, indoleamine 2,3- dioxygenase 1; overall survival, OS; progression-free survival, PFS.

We performed survival analysis based on IDO1 expression in 102 patients treated with ICIs in the first-line setting. OS, defined as time from initiation of ICIs to the time of death or last follow-up, and progression-free survival (PFS), defined as time from initiation of ICIs to the time of progression, death, or last follow-up, were both compared between the high and intermediate/low IDO1 groups (Figure 5). In the front-line setting, both PFS (HR = 0.49, 95% CI: 0.28–0.85, *p* = 0.010) and OS (HR = 0.33, 95% CI: 0.16–0.68, *p* = 0.001) were significantly longer in the high IDO1 group than in the intermediate/low IDO1 group (Figure 5: panels A and B). In contrast, no significant differences in PFS (HR = 0.69, 95% CI: 0.41–1.18, *p* = 0.175) or OS (HR = 0.75, 95% CI: 0.39–1.43, *p* = 0.382) were observed between IDO1 expression groups among patients treated with ICIs in the second-line and beyond setting (Figure 5, panels C and D). Importantly, high IDO1 was selected as a significant independent predictor of longer OS and PFS in multivariable analysis for the 102 patients treated with ICIs in the front-line setting (Tables 2 and 3).

**Table 2:** Multivariate analysis of overall (OS) survival from the time of initiation of front-line immune checkpoint inhibitor treatment (*n* = 102 patients)

	Univariate (*n* = 102)			Multivariate with TMB (cutoff: 10 mut/mb)^a^ (*n* = 83)			Multivariate with TMB (cutoff: 20 mut/mb)^a^ (*n* = 83)			Comment
Factor (*n* = 102 patients)	HR	95% CI	p value	HR	95% CI	p value	HR	95%CI	*p* value	
Age, >60 (years, *n* = 57)	0.78	0.47–1.30	0.338	–	–	–	–	–	–	
Sex, male (*n* = 46)	1.28	0.77–2.11	0.340	–	–	–	–	–	–	
IDO1, high (*n* = 27)	0.33	0.16–0.67	**0.001**^a^	0.31	0.11–0.87	**0.026**^a^	0.25	0.09–0.71	**0.009**^a^	**Higher IDO1 was associated with longer OS**
PD-1, high (*n* = 21)	0.43	0.20–0.95	**0.031**^a^	0.93	0.24–3.65	0.921	0.87	0.23–3.32	0.840	
PD-L1, high (*n* = 13)	0.24	0.06–1.00	**0.034**^a^	0.52	0.06–4.32	0.549	0.52	0.06–4.29	0.544	
PD-L2, high (*n* = 17)	0.31	0.11–0.86	**0.017**^a^	0.51	0.12–2.19	0.364	0.52	0.12–2.22	0.378	
CTLA-4, high (*n* = 20)	0.38	0.16–0.89	**0.020**^a^	0.99	0.32–3.02	0.980	1.02	0.33–3.12	0.974	
LAG-3, high (*n* = 22)	0.32	0.14–0.70	**0.003**^a^	0.54	0.21–1.39	0.193	0.62	0.24–1.60	0.322	
CD8, high (*n* = 23)	0.59	0.30–1.16	0.120	–	–	–	–	–	–	
Uterine (*n* = 6)	0.62	1.19–1.99	0.417	–	–	–	–	–	–	
Ovarian (*n* = 6)	0.66	0.21–2.12	0.488	–	–	–	–	–	–	
Lung (*n* = 3)	3.9e-8	0 - Inf	0.146	–	–	–	–	–	–	
Breast (*n* = 8)	0.58	1.18–1.85	0.351	–	–	–	–	–	–	
Colorectal (*n* = 28)	1.83	1.08–3.13	**0.024**^b^	1.54	0.81–2.92	0.190	1.44	0.77–2.69	0.248	
Sarcoma (*n* = 6)	0.65	0.20–2.08	0.465	–	–	–	–	–	–	
Gastric (*n* = 7)	0.91	0.33–2.51	0.858	–	–	–	–	–	–	
Pancreatic (*n* = 10)	1.99	0.89–4.42	**0.087**	1.92	0.69–5.35	0.214	1.92	0.71–5.19	0.200	
TMB, high (≥10 vs. < 10 mutation/mb, *n* = 15/83)	0.36	0.15–0.86	**0.016**^a^	0.53	0.21–1.31	0.168	–	–	–	
TMB, high (≥20 vs. < 20 mutation/mb, *n* = 5/83)	1.1e-08	0 - Inf	**0.006**	–	–	–	1.2e-08	0 - Inf	0.996	Only 5 patients had TMB ≥20 mutation/mb
MSI, high (*n* = 4/88)	0.19	0.03–1.38	**0.067**	–	–	–	–	–	–	

“High” expression of listed factors in this table means transcriptome expression rank values ranked from 75 to 100 on 1 to 100 scale. 102 patients treated with front-line immune checkpoint inhibitors were analyzed in the multivariate analysis. Multivariable analyses were performed in two ways: first analysis was performed with TMB (≥10 mutations/mb vs. <10) but without MSI; second analysis was performed with TMB (≥20 mutations/mb < 20) but without MSI. MSI was not included in multivariable analysis due to a small sample size. Only variables that showed significance in univariate analysis (*p* ≤ 0.1) were included in the multivariate analysis. Univariate analysis was performed for selected immune factors, age, sex, and selected cancer types with 20 or more patients in the entire cohort (*n* = 514) using log-rank test and HR was calculated using the cox hazard model. Multivariate analysis was performed using the Cox hazard model. Factors with *p* values of ≤ 0.1 in univariate analysis and ≤ 0.05 in multivariable analysis are shown in bold.

95% CI, 95% confidence interval; HR, hazard ratio; IDO1, indoleamine 2,3- dioxygenase 1; MSI, microsatellite instability; PD-1, programmed cell death protein 1; PD-L1, programmed death-ligand 1; TMB, tumor mutational burden.

a Statistically significant with better outcome in favor of the listed factors.

b Statistically significant with worse outcome with the listed factors.

**Table 3:** Multivariate analysis of progression-free survival from the time of initiation of front-line immune checkpoint inhibitor treatment (*n* = 102 patients)

PFS	Univariate (*n* = 102)			Multivariate with TMB (cutoff: 10 mut/mb)^a^ (*n* = 83)			Multivariate with TMB (cutoff: 20 mut/mb)^a^ (*n* = 83)			
Factor (*n* = 102 patients)	HR	95%CI	*p* value	HR	95%CI	*p* value	HR	95%CI	*p* value	
Age, >60 (*n* = 57)	0.68	0.43–1.06	**0.089**	0.84	0.50–1.41	0.507	0.75	0.45–1.26	0.272	
Sex, male (*n* = 46)	1.11	0.71–1.74	0.641	–	–	–	–	–	–	
IDO1, high (*n* = 27)	0.49	0.29–0.85	**0.012**^a^	0.44	0.20–0.99	**0.047**^a^	0.35	0.15–0.80	**0.013**^a^	**Higher IDO1 was associated with longer PFS.**
PD-1, high (*n* = 21)	0.59	0.32–1.07	0.075	0.87	0.30–2.57	0.802	0.87	0.30–2.54	0.802	
PD-L1, high (*n* = 13)	0.82	0.41–1.65	0.574	–	–	–	–	–	–	
PD-L2, high (*n* = 17)	0.52	0.26–1.04	**0.061**	0.54	0.18–1.57	0.256	0.53	0.18–1.54	0.242	
CTLA-4, high (*n* = 20)	0.50	0.27–0.96	**0.037**^a^	1.03	0.40–2.64	0.947	1.02	0.30–2.60	0.961	
LAG-3, high (*n* = 22)	0.55	0.31–0.98	**0.042**^a^	0.83	0.38–1.80	0.638	1.00	0.46–2.16	0.997	
CD8, high (*n* = 23)	0.74	0.42–1.28	0.272	–	–	–	–	–	–	
Uterine (*n* = 6)	0.67	0.24–1.82	0.420	–	–	–	–	–	–	
Ovarian (*n* = 6)	0.72	0.26–1.98	0.531	–	–	–	–	–	–	
Lung (*n* = 3)	0.42	0.06–2.99	0.365	–	–	–	–	–	–	
Breast (*n* = 8)	0.92	0.40–2.11	0.839	–	–	–	–	–	–	
Colorectal (*n* = 28)	1.35	0.82–2.22	0.239	–	–	–	–	–	–	
Sarcoma (*n* = 6)	0.81	0.33–2.02	0.657	–	–	–	–	–	–	
Gastric (*n* = 7)	0.73	0.27–1.99	0.536	–	–	–	–	–	–	
Pancreatic (*n* = 10)	2.39	1.17–4.86	**0.014**^b^	1.81	0.79–4.16	0.162	1.85	0.81–4.19	0.142	
TMB, high (≥10 vs. < 10 mutation/mb, *n* = 15/83)	0.41	0.18–0.87	**0.017**^a^	0.50	0.23–1.10	0.086	–	–	–	
TMB, high (≥20 vs. < 20 mutation/mb, *n* = 5/83)	1.1e-08	0 – Inf	**0.002**^a^	–	–	–	9.6e-09	0 - Inf	0.995	Only 5 patients had TMB ≥20 mutation/mb
MSI, high (*n* = 4/88)	0.14	0.02–1.05	**0.026**^a^	–	–	–	–	–	–	

“High” expression of listed factors in this table means transcriptome expression rank values ranked from 75 to 100 on 1 to 100 scale. 102 patients treated with front-line immune checkpoint inhibitors were analyzed in the multivariate analysis. Multivariable analyses were performed in two ways: first analysis was performed with TMB (≥10 mutations/mb versus <10) but without MSI; second analysis was performed with TMB (≥20 mutations/mb < 20) but without MSI. MSI was not included in multivariable analysis due to a small sample size. Only variables that showed significance in univariate analysis (*p* ≤ 0.1) were included in the multivariate analysis. Univariate analysis was performed for selected immune factors, age, sex, and selected cancer types with 20 or more patients in the entire cohort (*n* = 514) using log-rank test and HR was calculated using the cox hazard model. Multivariate analysis was performed using the Cox hazard model. Factors with *p* values of ≤ 0.1 in univariate analysis and ≤ 0.05 in multivariable analysis are shown in bold.

95% CI, 95% confidence interval; HR, hazard ratio; IDO1, indoleamine 2,3- dioxygenase 1; MSI, microsatellite instability; PD-1, programmed cell death protein 1; PD-L1, programmed death-ligand 1; TMB, tumor mutational burden.

a Statistically significant with better outcome in favor of the listed factors.

b Statistically significant with worse outcome with the listed factors.

High PD-1 or PD-L1 RNA expression predicted better OS (but not PFS) in the front-line setting in univariate but not in multivariate analysis, where only high IDO1 was independently predictive of longer PFS and OS in multivariate analysis (Tables 2 and 3; Figure S3) (though the small number of patients with high levels in this subgroup might have precluded robust statistical analysis). High TMB ≥10 mutations/mb vs. < 10, as well as ≥ 20 mutations/mb vs. < 20 predicted both longer PFS and OS in the univariate (but not multivariate) analysis (though only five patients had TMB ≥20, precluding robust statistical analysis of this subgroup) (Tables 2 and 3).

Finally, including both PD-L1 and IDO1 or both PD-1 and IDO1 transcript expression in the analysis of 102 patients treated with ICIs in the first-line setting further stratified OS outcome, with the high PD-1/high IDO1 group doing especially well compared to other groups (*p* = 0.012 for PD-L1/IDO1 and *p* = 0.008 for PD-1/IDO1—Figure S4; Table S3). This combined effect for PD-L1/IDO1 and PD-1/IDO1 was not seen when PFS was examined (Figure S5; Table S4). The latter findings were not surprising since, as mentioned earlier, neither PD-L1 or PD-1 RNA expression stratified PFS when examined alone, while both PD-L1 and PD-1 stratified OS in the front-line ICI setting in univariate analysis (Tables 2 and 3).

## Discussion

Although some proportion of patients achieve durable responses to ICIs, most people with cancer either develop primary or acquired resistance to immunotherapy and, therefore, the mechanisms of resistance need to be elucidated in order to improve therapeutic strategies.^20^^,^^21^ IDO1, an enzyme involved in tryptophan catabolism, has been recognized as a notable factor in creating an immunosuppressive TME that could contribute to ICI resistance.^3^^,^^22^ However, despite ample evidence from preclinical studies, little clinical benefit has been observed with IDO1 inhibitors either as monotherapy or in combination with an ICI. Thus, understanding interactions between IDO1 and other immune factors is essential in order to leverage IDO1 in developing novel immunotherapy strategies.

In the current study, IDO1 RNA expression was found to vary across tumor types and, in particular, uterine and ovarian cancers had high levels of IDO1 RNA expression. Historically, IDO1 positivity was mainly determined by immunohistochemistry (IHC), and the IDO1 expression in several types of cancer based on IHC varied across multiple studies. Most trials evaluating IDO1 inhibitors or modulators have not paid attention to IDO1 expression; it is therefore of potential interest that high IDO1 expression differs from patient to patient between and within tumor types.^8^ Results in the present work suggest that immunomic profiles need to be tested in individual tumors because, even though some patterns for expression emerged such as the higher proportion of gynecologic malignancies that are high IDO1 expressors, the heterogeneity between individual tumors means that individual testing is necessary.

Interrogation of the tumor immune microenvironment allows capturing dynamic interactions between IDO1 and other immune markers. We observed that high expression of factors related to the JAK-STAT pathway and immune checkpoints was particularly associated with high IDO1 RNA expression. IDO1 expression is normally regulated by IFNγ, TGF-β, and other cytokines through multiple pathways such as the JAK-STAT1, NF-κB, and PI3K pathways.^2^^,^^8^^,^^23^^,^^24^^,^^25^ Additionally, a study reported a signaling loop involving IL-6, STAT3, and the AhR that leads to sustained expression of IDO1 in the TME.^26^ Our study revealed that high expression of STAT1, an important component of the JAK-STAT signal, and high PD-L1, a critical immune checkpoint ligand, were independently and significantly associated with high IDO1 expression, and the correlation in our dataset was confirmed by analyzing TCGA data, which demonstrated a linear association between IDO1 and STAT1 and between IDO1 and PD-L1.^27^

Associations between IDO1 expression and mutations/alterations of oncogenes or tumor suppressor genes were previously documented in a study reporting that upregulation of IDO1 correlated with *PIK3CA E545K/R88Q* mutations.^28^ The present study validated an association between *PIK3CA* alterations and IDO1 high expression. Interestingly, a prior report suggests that the presence of *PIK3CA* mutations correlates with better outcome after ICI therapy, at least in gastric cancer patients.^29^ Our analysis also revealed an association between IDO1 expression and *CTCF* alterations. *CTCF* encodes the chromatin organizing protein CTCF, which is an 11-ZF DNA binding protein with a variety of functions to regulate chromatin structures. It can act as a tumor suppressor, and loss-of-function alterations have been reported to be pro-tumorigenic in endometrial/uterine carcinoma.^30^

As mentioned, strong correlations were observed between high IDO1 expression and immune checkpoints such as PD-L1, as well as markers related to MDSCs, tumor-associated macrophages, and regulatory T cells. In preclinical studies, increased expression of IDO1 was associated with increased levels of regulatory T cells, MDSCs, and upregulation of PD-1.^7^^,^^8^^,^^31^ In parallel, nivolumab (PD-1 inhibitor) was reported to upregulate the expression of IDO1, suggesting the possibility of PD-1 inhibitors to overcome immunosuppressive effect driven by IDO1 in TME, and an essential role of IDO1 to regulate the downstream PD-1 expression.^32^ A study examining the expression of IDO1, PD-L1, and FOXP3-positive regulatory T cells in the melanoma tumor cells showed that these upregulations were driven by CD8^+^ T cells. IDO1 and PD-L1 were upregulated via IFNγ, and Tregs were upregulated by CCR4-binding chemokines, suggesting that these immunosuppressive factors may be a therapeutic target within a pre-existing T cell-inflamed TME.^23^ A phase 1/2 study evaluating an immunomodulatory vaccine against IDO and PD-L1 combined with nivolumab (anti-PD-1 ICI) demonstrated high ORR (80%) in metastatic malignant melanoma, suggesting that targeting both IDO1 and PD-L1/PD-1 is a potential strategy to enhance the efficacy of immunotherapy.^33^ In addition to PD-L1/PD-1 inhibition, a preclinical study suggests a synergistic effect of CTLA-4 and IDO1 blockade by inducing an increase in effector T cell to regulatory T cell ratio. IDO1 is associated with upregulation of regulatory T cells and its blockade appears to trigger a potent effector T cell response primed by CTLA-4 antibody.^22^ The role of AhR, a receptor of tryptophan catabolites, could also link expression of IDO1 and other markers observed in our analysis. AhR has a role in upregulating IDO1, forming an AhR-IDO1-AhR ligand amplification loop, creating an immunosuppressive TME.^34^^,^^35^ Kynurenine, a metabolite of tryptophan through IDO1, induces PD-1 on tumor-infiltrating CD8^+^ T cells.^5^ AhR also plays a central role in IFNγ-induced expression of IDO1, PD-L1, CTLA-4, LAG-3, and CD39 in an oral squamous cell carcinoma model.^36^ These imply the need for further research focusing on tryptophan metabolites and their impact on the efficacy of ICIs. Our findings support a link between IDO1 and immune checkpoints in the TME, warranting further clinical evaluation to target both to improve the efficacy of antineoplastic therapies.

The search for an appropriate biomarker to predict response to ICIs in patients with cancer is an ongoing and unsolved but important topic. Several factors such as PD-L1 expression, TMB, and tumor-infiltrating lymphocytes have been proposed as predictive markers for the efficacy of ICIs.^37^^,^^38^ Additionally, positive PD-1 expression on tumor-infiltrating lymphocytes may predict a longer survival after PD-1/PD-L1 inhibitors.^39^ Our current work demonstrated that high IDO1 RNA expression was associated with longer PFS and OS in patients treated with front-line ICIs (but not in later line settings). High IDO1 also did not have a prognostic impact on OS in immunotherapy-naive patients. We found that PD-L1 and PD-1 expression may further stratify the predictive effect of IDO1 expression in the context of OS in the front-line ICI-treated patients. These observations suggest that high IDO1 could be a predictive biomarker for the efficacy of ICI treatment, particularly in the first-line setting. On first impression, this result is puzzling as historically, IDO1 expression in the TME was proposed as an immunosuppressive factor potentially resulting in resistance to ICIs. When IDO1 activity is expressed as kynurenine to tryptophan ratio, higher IDO activity was associated with shorter PFS and OS in 26 patients with non-small-cell lung carcinoma (NSCLC) treated with nivolumab monotherapy.^40^ Additionally, one study comprising 41 patients with NSCLC treated with second-line ICIs revealed that pretreatment IDO1 mRNA expression was lower in 16 responders than in 25 non-responders.^41^ However, the small number of patients in the prior studies and the fact that second-line ICIs were examined, while our results showing that high IDO1 expression was associated with longer survival pertained only to ICIs given to patients in the front-line setting, and not the second-line or beyond setting, indicate that the prior and current results may not be contradictory. Furthermore, a different study in 67 patients with NSCLC showed that high PD-L1 and high IDO1 correlated independently in multivariate analysis with higher response rates (objective response rate was 87.5% if both positive, 60% if one of them was positive, 22.7% if both negative; only IDO1 correlated with PFS, at least in the front-line ICI setting).^42^ Even so, the reasons for our findings as well as those of the prior study mentioned earlier are unclear, since high IDO1 is related to an immunosuppressive TME. Of potential relevance, recent data suggest that the upregulation of IDO1 could be driven by tumor-infiltrating CD8^+^ T cells, which in turn suggests that IDO1 expression may be induced in the presence of a pre-existing “hot” environment, which makes ICIs more effective.^23^ This is supported by our analysis showing a correlation between high IDO1 and high CXCL10; the latter IFN-γ-related chemokine has a pivotal influence on cytotoxic T cell tumor recruitment and ICI response.^43^^,^^44^ Furthermore, high IDO1 correlated significantly with high STAT1 in our study, and STAT proteins play indispensable roles in cytokine signaling and T helper cell differentiation; specifically, STAT1 plays a vital role in interferon signaling, which initiates the expression of genes encoding proteins with antitumor and apoptotic roles.^45^

In summary, we observed that IDO1 transcript expression varies substantially between and within cancer types, with uterine and ovarian cancers having the highest proportion of high expressors and hepatobiliary cancers having no high expressors, albeit with a limited number of patients tested. High IDO1 expression often co-segregated with high expression of immune checkpoints across cancers and there was an independent, statistically significant, and linear relationship between IDO1 expression and that of PD-L1, STAT1, and CXCL10 transcripts. IDO1 expression level was not a prognostic factor for survival from the time of advanced/metastatic disease in immunotherapy-naive patients, nor did IDO1 expression level predict outcome after ICI therapy in the second-line setting or beyond. However, counter-intuitively, high IDO1 expression did predict longer PFS and OS after first-line ICI therapy. These data suggest that there is a more complex mechanism of action for IDO1 than previously appreciated, perhaps because of its association with CD8^+^ T cell infiltration in a “hot” microenvironment or the correlation between high IDO1 and high STAT1, a molecule important for amplifying cancer immunity^46^; the correlation between IDO1 and CXCL10 may also be important because the latter acts to promote T cell infiltrates. Including both PD-L1 and IDO1 or both PD-1 and IDO1 transcript expression in the analysis of front-line ICI-treated patients further stratified survival (but not PFS) outcome, with the high PD-1/high IDO1 group doing especially well compared to other groups. Future studies to prospectively address the predictive power of IDO1 expression after immunotherapy are needed, as is in-depth biologic interrogation to better understand why high IDO1 expression correlates with improved immunotherapy outcomes in the front-line setting.

### Limitations of the study

There are several important limitations to our study. First, our use of transcriptomics did not permit analysis of cell type expression; such analysis is important for future studies. Several gene signatures have been associated with IDO1 pathway activation^47^; transcriptomic data about these gene signatures were not available to us but merit investigation in future studies. Second, the clinical correlates and outcomes were examined in the real world, and additional prospective studies are warranted. Furthermore, clinical factors such as comorbidities and medications that may affect the expression of immunoregulatory factors, including IDO1, were not taken into account. In addition, while interrogating a pan-cancer cohort might point to generalizability of the data across malignancies, there were not adequate numbers of patients in most individual cancer types to perform an in-depth histology-based analysis that might uncover differences between cancer types.

## STAR★Methods

### Key resources table

**Table:** 

REAGENT or RESOURCE	SOURCE	IDENTIFIER
**Biological samples**		

Tumor tissue from patients with solid tumors	Center for Personalized Cancer Therapy, Moores Cancer Center, University of California San Diego	N/A (This paper)
Blood samples from patients with solid tumors	Center for Personalized Cancer Therapy, Moores Cancer Center, University of California San Diego	N/A (This paper)

**Critical commercial assays**		

FoundationOne CDx	Foundation Medicine	https://www.foundationmedicine.com/test/foundationone-cdx
FoundationOne Liquid CDx	Foundation Medicine	https://www.foundationmedicine.com/test/foundationone-liquid-cdx
Tempus xT CDx	Tempus	https://www.tempus.com
OmniSeq INSIGHT	Labcorp Oncology (OmniSeq)	https://oncology.labcorp.com/os-welcome
Oncomine Immune Response Research Assay	Thermo Fisher Scientific, Waltham, MA	https://www.thermofisher.com/us/en/home/clinical/preclinical-companion-diagnostic-development/oncomine-oncology/oncomine-immune-response-research-assay.html
truXTRAC FFPE Extraction Kit	Covaris, Inc., Woburn, MA	http://www.covaris.com
Quant-iT RNA HS Assay	Thermo Fisher Scientific, Waltham, MA	https://www.thermofisher.com/us/en/home.html
Ion Torrent S5Xl system, RNA sequencing absolute reads were generated using the immuneResponseRNA (v5.2.0.0) plug-in of the Torrent Suite Software	Thermo Fisher Scientific, Waltham, MA	https://www.thermofisher.com/us/en/home.html

**Deposited data**		

Clinical trial registry number	ClinicalTrials.gov	NCT02478931
The Cancer Genomic Atlas (TCGA)	National Cancer Institute CDC Data Portal	https://portal.gdc.cancer.gov/
cBioPortal for Cancer Genomics	Center for Molecular Oncology at Memorial Sloan Kettering Cancer Center	https://www.cbioportal.org/

**Software and algorithms**		

R programming language, v4.3.2	R Core Team	https://www.R-project.org/
R package, survminer, v 0.4.9	Survival analysis	https://cran.r-project.org/web/packages/survminer/index.html
R package, survival v3.5-7	Survival analysis	https://cran.r-project.org/web/packages/survival/index.html
R package, ggplot2, v3.5.0	Plot, tidyverse toolkit	https://cran.r-project.org/web/packages/ggplot2/index.html
R package, tableone, v0.13.2	Formatting, baseline characteristics analysis	https://cran.r-project.org/web/packages/tableone/index.html
R package, epiDisplay, v3.5.0.2	Formatting, logistic regression tool	https://cran.r-project.org/web/packages/epiDisplay/index.html
Microsoft Excel, Microsoft 365	Microsoft, Redmond, WA	https://www.microsoft.com/en-us/microsoft-365/excel
Morpheus	Broad Institute, Cambridge, MA	https://software.broadinstitute.org/morpheus
BioRender	A graphical abstract was created with BioRender.com.	https://www.biorender.com/

**Other**		

Clinical information of enrolled patients	Center for Personalized Cancer Therapy, Moores Cancer Center, University of California San Diego	N/A (This paper)

### Resource availability

#### Lead contact

Further information and requests for resources and reagents should be directed to and will be fulfilled by the lead contact, Yu Fujiwara (yu.fujiwara@roswellpark.org).

#### Materials availability

This study did not generate new unique reagents.

#### Data and code availability

• The tumor RNA sequencing percentile rank data and de-identified patient clinical data in this paper will be shared by lead author upon request. This paper also analyzes existing, publicly available data. These accession numbers for the datasets are listed in the key resources table.
• This paper does not report original code. No customized code was used in the present study. R packages used for data analysis and output were publicly and freely available online and listed in the key resources table. Corresponding R packages were used for data analysis, as described in detail in the Quantification and statistical analysis section below.
• Any additional information required to reanalyze the data reported in this paper is available from the lead contact upon request.

### Experimental model and study participant details

#### Ethical oversight, patient consent and enrollment

In total, 514 patients with solid tumors at the Center for Personalized Cancer Therapy, Moores Cancer Center, University of California San Diego, participated in this study. This study followed the guidelines of the IRB-approved UCSD- Profile Related Evidence Determining Individualized Cancer Therapy (PREDICT) study (NCT02478931, https://clinicaltrials.gov/ct2/show/NCT02478931) and any investigational studies for which the patients gave consent.^8^^,^^48^^,^^49^^,^^50^ These patients were diagnosed with any types of solid tumors and their age ranged from 23 to 93 years old. Overall, 204 patients were male, and 310 patients were female. The study cohort comprised individuals identifying as White, Black, Native American, Asian, Pacific Islander, and others, ensuring a wide representation of demographic groups in this research.

The PREDICT study aims to learn about personalized cancer therapy including response to treatment by evaluating genetic makeup through genomic and transcriptomic testing results of enrolled patients. Medical records were examined for results of molecular profiling obtained through standard of care testing to help understand how these test results would affect and predict response to therapy.

The purpose of the present study is to elucidate the mRNA expression of IDO1 based on cancer types, and its association with other immunoregulatory factors related to the IDO1 pathway and mechanisms of resistance to ICIs. This analysis was performed using percentile data of mRNA expression defined below of all 514 patients enrolled in the study. To further examine the role of IDO1 in ICI therapy, survival such as PFS and OS was evaluated in patients who received front-line ICI therapy (n = 102) based on IDO1 transcriptome expression, and multivariable analyses were performed to evaluate other relevant factors that could affect survival outcomes.

### Method details

#### Tissue collection and transcriptome sequencing

Anatomical Pathologist qualified formalin-fixed, paraffin-embedded (FFPE) tumor specimens were evaluated with RNA transcriptome sequencing of a clinically validated gene expression panel relating to the anticancer immune response as previously described.^51^ Total RNA was extracted using the truXTRAC FFPE Extraction Kit (Covaris, Inc., Woburn, MA), eluted in 50 μL water with yield determined by utilizing the Quant-iT RNA HS Assay (Thermo Fisher Scientific, Waltham, MA) according to the manufacturer’s recommendations. A predefined yield of 10 ng RNA was considered acceptable to ensure library preparation. Following sequencing on an Ion Torrent S5Xl system, RNA sequencing absolute reads were generated using the immuneResponseRNA (v5.2.0.0) plug-in of the Torrent Suite Software (Thermo Fisher Scientific, Waltham, MA).

#### Calculating percentile RNA rank by using an independent reference cohort of 735 tumors

Immune factors related to IDO1, tryptophan catabolism, and immune checkpoints were selected for evaluation as follows: CD38, CD39 (adenosine pathway), VEGF-A (angiogenesis), AXL, TGFB1 (EMT: epithelial-mesenchymal transition), CD80, CD86, CTLA-4, LAG-3, PD-1, PD-L1, PD-L2, TIGIT, TIM3, VISTA (inhibitory checkpoints), IDO1, IDO2, TDO2 (tryptophan-IDO-kynurenine pathway), IFNγ, IL-6, STAT1, STAT3 (IFNγ-JAK-STAT pathway), CCR1, CCR2, CXCR2 (myeloid-derived suppressive cell), AKT1, MTOR, PIK3CA (mTOR pathway), CD28, CD40, GITR, ICOS, ICOSLG, OX40, OX40L (co-stimulatory checkpoints), CSF1R, CXCR4 (tumor-associated macrophage), CD8, CXCL9, and CXCL10 (cytotoxic T cell), CCR4, CCR5, and FOXP3 (regulatory T cell) (Resource S1). The RNA expression of these selected immune factors was calculated and, then, the transcript abundance of these molecules was normalized and compared to the internal reference consisting of 735 tumors spanning 35 histologies. Rank values of each selected factor were determined on a scale of 1–100 as previously reported.^51^ Rank values were categorized as low [0–24], intermediate [25–74], and high [75–100]. The frequency of high, intermediate, and low IDO1 RNA expression by primary site of cancer was calculated and plotted on a bar graph. To visualize expression of selected inhibitory checkpoints (PD-1, PD-L1, PD-L2, CTLA-4, and LAG-3) according to IDO1 expression, a heatmap was generated with edits using the Morpheus software (https://software.broadinstitute.org/morpheus). Microsatellite instability status (MSI) and tumor mutational burden (TMB), which are important immunotherapy predictors, were assessed in 440 and 392 patients, respectively, using previously reported techniques, respectively.^38^^,^^52^^,^^53^^,^^54^ Two different cutoff numbers (≥10 vs. < 10 and ≥20 vs. < 20 mutations/mb) were used to classify TMB into high and low groups.

#### Analysis of selected immune factors based on Ido1 RNA expression

The association between IDO1 RNA expression and factors including RNA expression of selected immune molecules (high [≥75^th^ percentile RNA rank] vs. intermediate/low [<75^th^ percentile RNA rank]), clinical characteristics such as age, sex, MSI (high vs. stable), TMB (high vs. low), and cancer type were assessed. Odds ratios (ORs) for high IDO1 expression were calculated based on these factors using the chi-squared test for univariate analysis. Then, multivariate analysis was performed to calculate adjusted ORs using logistic regression. Four models with and without MSI status and/or TMB with two different cutoff numbers defined above were adopted to perform multivariate analysis. We used the entire cohort (n = 514) to perform these analyses.

### Quantification and statistical analysis

#### Analysis of genomic alteration frequency based on Ido1 RNA expression

Comprehensive genomic profiling information included reference laboratory clinical-grade next generation sequencing (NGS) testing from Foundation Medicine (https://www.foundationmedicine.com/test/foundationone-cdx), Tempus https://www.tempus.com) or OmniSeq (https://oncology.labcorp.com/os-welcome) in 464 out of 514 enrolled patients. To characterize the alteration landscape based on the IDO1 RNA expression groups defined above, the alteration frequency of each detected gene was calculated in IDO1 high and intermediate/low groups, respectively. The top 20 most commonly detected alterations in each IDO1 expression group were compared and visualized in a bar graph, and eventually, 34 genes were included for this analysis after removing duplicated genes in both IDO1 expression groups. The alteration frequency of each group was then statistically compared using Fisher’s exact test with a correction with Bonferroni method (correction of multiple testing), and thus, p < 0.001471 (=0.05/34) was considered statistically different.

#### TCGA dataset and analysis

To externally validate the findings on the association of RNA expression between IDO1 and other immune molecules, pan-cancer data from TCGA dataset for 1210 samples with mRNA expression information were accessed and analyzed. Data were accessed and figures were created through cBioPortal for Cancer Genomics (https://www.cbioportal.org/). The correlation between mRNA expression of IDO1 and immune factors of interest was evaluated using Spearman test. We defined a relatively strong correlation when the Spearman correlation was 0.5 and higher.

#### Survival outcome in immune checkpoint inhibitor (ICI) treated participants

Survival outcomes were analyzed based on IDO1 expression in patients treated with ICIs in the first line (n = 102) and second line and beyond setting (n = 115). We also performed survival analysis from date of advanced/metastatic diagnosis in patients with advanced cancer who were not treated with ICIs (n = 272). Patients were divided into high IDO1 (≥75th percentile RNA rank) and intermediate/low (<75th percentile RNA rank) IDO1 groups as defined above. Patient characteristics of those treated with front-line ICIs including age, sex, tumor stage at cancer diagnosis, cancer type, cancer status at mRNA expression analysis, MSI status, and TMB were summarized. Progression-free survival (PFS) was defined from the time of treatment initiation to the time of progression, death, or last follow up date, and overall survival (OS) was defined from the time of treatment initiation to the time of death or last follow up date. Patients still progression-free or alive at last follow up were censored on that date for PFS and OS, respectively. For the 272 patients not treated with ICI, OS stratified by high vs. medium/low IDO1 level was also assessed by the Kaplan-Meier method, with the start date being date of advanced/metastatic disease. Survival probability was calculated using the Kaplan-Meier estimator and the long-rank test was used to compare survival between groups.

These survival analyses were performed by stratifying patients into high versus intermediate/low expression for IDO1, PD-L1, and PD-1 and exploring outcomes by single marker and combined marker status. In all cases, high expression denotes ≥75th percentile RNA rank and intermediate/low denotes <75th percentile RNA rank; See STAR methods section above. Additionally, pre-specified variables known to potentially affect the survival outcomes in patients treated with ICIs including age, sex, IDO1, PD-1, PD-L1, PD-L2, CTLA-4, LAG-3, CD8, MSI, TMB, and having a cancer type with 20 or more patients in the overall cohort (uterine, ovarian, lung, breast, colorectal, gastric, pancreatic cancer, and sarcoma) were evaluated in both univariate and multivariate analysis using the Cox proportional hazards model for OS and PFS. These multivariable analyses were performed to compare and elucidate the factors that strongly correlate with survival outcomes. In multivariate analyses, factors with p value less than 0.1 in univariate analysis were chosen, and three models with and without TMB with two different cutoff numbers (≥10 mutations/mb vs. < 10 and ≥20 mutations/mb vs. < 20) defined above were used. Adjusted hazard ratio (HR) with 95% confidence interval (CI) was calculated for each variable with p value, and p values less than 0.05 in the multivariable analysis were considered significant. All statistical analyses were performed using R version 4.2.1 via RStudio (http://www.rstudio.com/).

### Additional resources

This study is part of a clinical trial registered on ClinicalTrials.gov (Identifier: NCT02478931, https://clinicaltrials.gov/ct2/show/NCT02478931).

## Supplemental information

Document S1. Figures S1–S5, Tables S1–S4, and Resource S1
