# Supplementary material for: High indoleamine 2,3-dioxygenase transcript levels predict better outcome after front-line cancer immunotherapy
Source: iScience. 2024 Mar 28;27(4):109632. doi: 10.1016/j.isci.2024.109632 (PMC11022045; doi:10.1016/j.isci.2024.109632)
Supplement: Document S1. Figures S1–S5, Tables S1–S4, and Resource S1 [file mmc1.pdf]

iScience, Volume *27*

Supplemental information

High indoleamine 2,3-dioxygenase transcript levels predict better outcome after front-line cancer immunotherapy

Yu Fujiwara, Shumei Kato, Daisuke Nishizaki, Hirotaka Miyashita, Suzanna Lee, Mary K. Nesline, Jeffrey M. Conroy, Paul DePietro, Sarabjot Pabla, Scott M. Lippman, and Razelle Kurzrock

Table S1. Clinical characteristics of all patients (n = 514), related to Table 1.

**Characteristics**	**n = 514**
**Age (median [Interquartile range])**	60.8 [50.5-69.5]
**Sex (number, %)**	
Male	204 (39.7)
Female	310 (60.3)
**Cancer type (number, %)**	
Colorectal cancer	140 (27.2)
Pancreatic cancer	55 (10.7)
Breast cancer	49 (9.5)
Ovarian cancer	43 (8.4)
Gastric cancer	25 (4.9)
Sarcoma	24 (4.7)
Uterine cancer	24 (4.7)
Lung cancer	20 (3.9)
Liver and bile duct cancer	19 (3.7)
Esophageal cancer	17 (3.3)
Neuroendocrine tumors	15 (2.9)
Cancer of unknown primary	13 (2.5)
Head and neck cancer	12 (2.3)
Small intestine cancer	12 (2.3)
Melanoma	6 (1.2)
Cervical cancer	5 (1.0)
Bladder cancer	4 (0.8)
Gallbladder cancer and extrahepatic cholangiocarcinoma	4 (0.8)
Prostate cancer	4 (0.8)
Central nervous system tumor	3 (0.6)
Renal cell carcinoma	3 (0.6)
Squamous cell carcinoma of the skin	3 (0.6)
Thyroid cancer	3 (0.6)
Adrenal gland cancer	2 (0.4)
Lipomatous neoplasm	2 (0.4)
Mesothelioma	2 (0.4)
Adrenal cortical carcinoma	1 (0.2)
Basal cell carcinoma of the skin	1 (0.2)
Ocular melanoma	1 (0.2)
Primary peritoneal carcinoma	1 (0.2)
Thymic Cancer	1 (0.2)

Table S2. Clinical characteristics of patients with advanced solid tumors treated with front-line immune checkpoint inhibitors (n = 102) based on IDO1 RNA expression, related to Table 1.

	First line immune checkpoint inhibitor (n=102)	IDO1		P value (High vs Intermediate/low IDO1)
		High	Intermediate/low	
Number (%)	102 (100)	27 (26.5)	75 (73.5)	
Age (median [IQR])	61.92 [54.38, 70.84]	61.82 [56.70, 70.75]	61.97 [53.99, 70.93]	0.776
Sex = Female/Male (%)	56/46 (54.9/45.1)	21/6 (77.8/22.2)	35/40 (46.7/53.3)	**0.007**
	More women treated with first line checkpoint inhibitors had high IDO1 expression.			
Primary site (%)				
Basal Cell Carcinoma	1 (100)	0 (0.0)	1 (100)	
Bladder Cancer	2 (100)	0 (0.0)	2 (100)	
Breast Cancer	8 (100)	4 (50.0)	4 (50.0)	
Cervical Cancer	1 (100)	1 (100)	0 (0.0)	
Colorectal Cancer	28 (100)	1 (3.6)	27 (96.4)	
Esophageal Cancer	5 (100)	1 (20.0)	4 (80.0)	
Gallbladder and Extrahepatic Bile Duct Cancers	1 (100)	0 (0.0)	1 (100)	
Head and Neck Cancer	1 (100)	0 (0.0)	1 (100)	
Liver and Bile Duct Cancer	7 (100)	0 (0.0)	7 (100)	
Lung Cancer	3 (100)	1 (33.3)	2 (66.7)	
Melanoma	2 (100)	2 (100)	0 (0.0)	
Mesothelioma	1 (100)	0 (0.0)	1 (100)	
Neuroendocrine Tumors	2 (100)	1 (50.0)	1 (50.0)	
Ovarian Cancer	6 (100)	4 (66.7)	2 (33.3)	
Pancreatic Cancer	10 (100)	1 (11.1)	9 (88.9)	
Prostate Cancer	1 (100)	0 (0.0)	1 (100)	
Sarcoma	6 (100)	2 (33.3)	4 (66.7)	
Stomach Cancer	7 (100)	1 (14.3)	6 (85.7)	
Thyroid Cancer	1 (100)	1 (100)	0 (0.0)	
Unknown Primary Cancer	3 (100)	2 (66.7)	1 (33.3)	
Uterine Cancer	6 (100)	5 (83.3)	1 (16.7)	
Cancer status at mRNA expression analysis (%)				1
Metastatic	45 (100)	12 (26.7)	33 (73.3)	
Primary	55 (100)	15 (27.3)	40 (72.7)	
Recurrent	2 (100)	0 (0.0)	2 (100)	
TMB (median [IQR], mutation/mb)	5.20 [3.50, 7.90]	5.20 [3.40, 7.90]	5.30 [3.50, 7.82]	0.756

MSI status (%)				0.031
Stable	84 (100)	23 (27.4)	61 (72.6)	
MSI-H	4 (100)	3 (75.0)	1 (25.0)	
Not available	14 (100)	1 (7.1)	13 (92.9)	
	MSI high was more frequently observed in the high IDO1 expression group treated with 1st line checkpoint inhibitors, but the number of patients with MSI-high was small.			
ICI treatment				
PD-1 inhibitor	93 (100)	24 (25.8)	69 (74.2)	
ICI monotherapy	44 (100)	12 (27.9)	32 (72.1)	
Combination therapy	49 (100)	12 (24.5)	37 (75.5)	
PD-L1 inhibitor	4 (100)	1 (25.0)	3 (75.0)	
ICI monotherapy	2 (100)	1 (50.0)	1 (50.0)	
Combination therapy	2 (100)	0 (0.0)	2 (100)	
CTLA-4 inhibitor	5 (100)	2 (40.0)	3 (60.0)	
PD-1 + CTLA-4 inhibitors	5 (100)	2 (40.0)	3 (60.0)	

The RNA expression of the selected immune factors was calculated, and the transcript abundance of these molecules was normalized and compared to the internal reference consisting of 735 tumors spanning 35 histologies. Rank values of each selected factor were determined on a scale of 1 to 100 as previously reported. Rank values were categorized as low [0-24], intermediate [25-74], and high [75-100].

Abbreviations: CTLA-4; cytotoxic T lymphocyte antigen-4; ICI, immune checkpoint inhibitor; IDO, indolamine-2,3-dioxygenase 1; MSI, microsatellite instability; mRNA, messenger ribonucleic acid; PD-1, programmed cell death protein 1; PD-L1, programmed cell ligand-1; TMB, tumor mutational burden.

Resource S1. The selected immune factors in the tumor microenvironment associated with indoleamine 2,3-deoxygenease and immune checkpoint inhibitors, related to STAR Methods.

Classification	Factor	Alternative name/abbreviation	Role in tumor immune microenvironment	Ref
Adenosine pathway	CD38	ADP-ribosyl cyclase/cyclic ADP-ribose hydrolase 1	Induces an immunosuppressive tumor microenvironment (TME) by increasing myeloid-derived suppressor cells (MDSCs) and regulatory T cells	(1)
	CD39	Ectonucleoside triphosphate diphosphohydrolase-1	Associated with decreased anti-tumor T cells and increased dendritic cells	(2)
Angiogenesis	VEGF-A	Vascular permeability factor	Decreases pro-inflammatory cytokines	(3,4)
Epithelial-mesenchymal transition	AXL	-	Induces epithelial-mesenchymal transition and T cell exhaustion	(5)
	TGFB1	Transforming growth factor beta 1	Decreases T cell infiltration by inducing fibrosis around tumor cells and attenuates response to immune checkpoint inhibitors by contributing to exclusion of T cells	(6,7)
Inhibitory immune checkpoint	CD80	B7-1	Inhibits the activation of T cell in the presence of CTLA-4	(8)
	CD86	B7-2	Inhibits the activation of T cell in the presence of CTLA-4	(8)
	CTLA-4	CD152	Downregulates costimulatory signals of effector T cells	(8)
	LAG-3	CD223	Competitively inhibits function of T cell receptor	(9,10)
	PD-1	CD279	Deactivates the function of effector T cell	(11,12)
	PD-L1	CD274, B7-H1	Inhibits the function of cytotoxic T cell by binding PD-1	(11,12)
	PD-L2	CD273, B7-DC	Inhibits the function of cytotoxic T cell by binding PD-1	(13)
	TIGIT	WUCAM, Vstm3	Induces T cell exhaustion and augment escape from cancer-immunity	(14)
	TIM3	CD366, HAVCR2	Associated with T cell and NK cell exhaustion	(15)
	VISTA	VSIR	Inhibits anti-tumor T cells and reduces T-cell cytokine production	(16).
Tryptophan-IDO-	IDO1	Indoleamine 2,3-Dioxygenase 1	Catabolizes tryptophan to its metabolites, resulting in	(17,18)

kynurenine pathway			immunosuppressive tumor microenvironment	
	IDO2	Indoleamine 2,3-Dioxygenase 2	Same as IDO1	(19-21)
	TDO2	Tryptophan 2,3-Dioxygenase	Same as IDO1	(21,22)
IFNγ-JAK-STAT pathway	IFNγ	Interferon-gamma	Stimulates anti-tumor immunity. Regulates downstream IDO1 expression	(19,23)
	IL-6	Interleukin-6	Activates STAT3 leading to IDO1 production	(24)
	STAT1	Signal Transducer And Activator Of Transcription 1	Upstream factor to produce IDO1	(21)
	STAT3	Signal Transducer And Activator Of Transcription 3	Produces IDO1 via the IFNγ and JAK pathway	(24)
Myeloid-derived suppressive cell	CCR1	CD191	Induces MDSCs in the TME	(25)
	CCR2	CD192	Induces MDSCs in the TME	(26)
	CXCR2	CD182, interleukin-8 receptor B	Promotes mesenchymal features in cancer cells	(27)
mTOR pathway	AKT1	v-akt Murine Thymoma Viral Oncogene Homolog 1	The PI3K-AKT-mTOR pathway induces the downstream expression of IDO1. Associated with the production of immunosuppressive cytokines such as VEGF and a decrease in tumor-infiltrating T cells	(28)
	MTOR	mechanistic target of rapamycin		
	PIK3CA	Phosphatidylinositol 4,5-bisphosphate 3-kinase catalytic subunit alpha isoform		
Co-stimulatory checkpoint	CD28	Tp44	Competes with CTLA-4 for the B7 ligand, facilitating T cell production	(8)
	CD40	TNFRSF5	A tumor necrosis factor receptor. Activates antigen-presenting cells and decreases MDSCs, leading to T-cell infiltration	(29)
	GITR	CD357	Helps T-cell memory function against tumor cells	(30)
	ICOS	CD278, AILIM, CRP-1, H4	Helps the function of T-cell memory to facilitate anti-tumor immunity	(31)
	ICOSLG	CD275, B7-H2	A ligand to ICOS	(31)

	OX40	CD134, TNFRSF4	A tumor necrosis factor receptor. Associated with the suppression of regulatory T cells and an increase in tumor-infiltrating lymphocytes	(32,33)
	OX40L	CD252	A ligand to OX40	(32,33)
Tumor-associated macrophage	CSF1R	CD115, M-CSFR	A receptor for macrophage-colony stimulating growth factor. Induces tumor-associated macrophages.	(34,35)
	CXCR4	CD184	A chemokine receptor enriched on tumor-associated macrophages, leading to tumor progression, angiogenesis, and metastasis.	(36,37)
Cytotoxic T cell	CD8	-	The primary measure of the anti-tumor immunity.	(38)
	CXCL9	Monokine induced by gamma interferon	Promotes tumor-infiltrating lymphocytes driven by IFN**γ**	(39,40)
	CXCL10	Interferon gamma-induced protein 10	Promotes tumor-infiltrating lymphocytes driven by IFN**γ**	(39,41)
Regulatory T cell	CCR4	CD194	Associated with a recruitment of regulatory T cells	(42)
	CCR5	CD195	Associated with a recruitment of regulatory T cells	(43)
	FOXP3	Scurfin	A major protein specifically expression on the surface of regulatory T cells	(44)

Immune factors related to IDO1, tryptophan catabolism, and immune checkpoints were selected for evaluation as follows: CD38, CD39 (adenosine pathway), VEGF-A (angiogenesis), AXL, TGFB1 (EMT: epithelial-mesenchymal transition), CD80, CD86, CTLA-4, LAG-3, PD-1, PD-L1, PD-L2, TIGIT, TIM3, VISTA (inhibitory checkpoints), IDO1, IDO2, TDO2 (tryptophan-IDO-kynurenine pathway), IFN**γ**, IL-6, STAT1, STAT3 (IFN**γ**-JAK-STAT pathway), CCR1, CCR2, CXCR2 (myeloid-derived suppressive cell), AKT1, MTOR, PIK3CA (mTOR pathway), CD28, CD40, GITR, ICOS, ICOSLG, OX40, OX40L (co-stimulatory checkpoints), CSF1R, CXCR4 (tumor-associated macrophage), CD8, CXCL9, CXCL10 (cytotoxic T cell), CCR4, CCR5, and FOXP3 (regulatory T cell).

Figure S1. TCGA data analysis of mRNA expression of IDO1, PD-L1, and STAT1, related to Table 1.

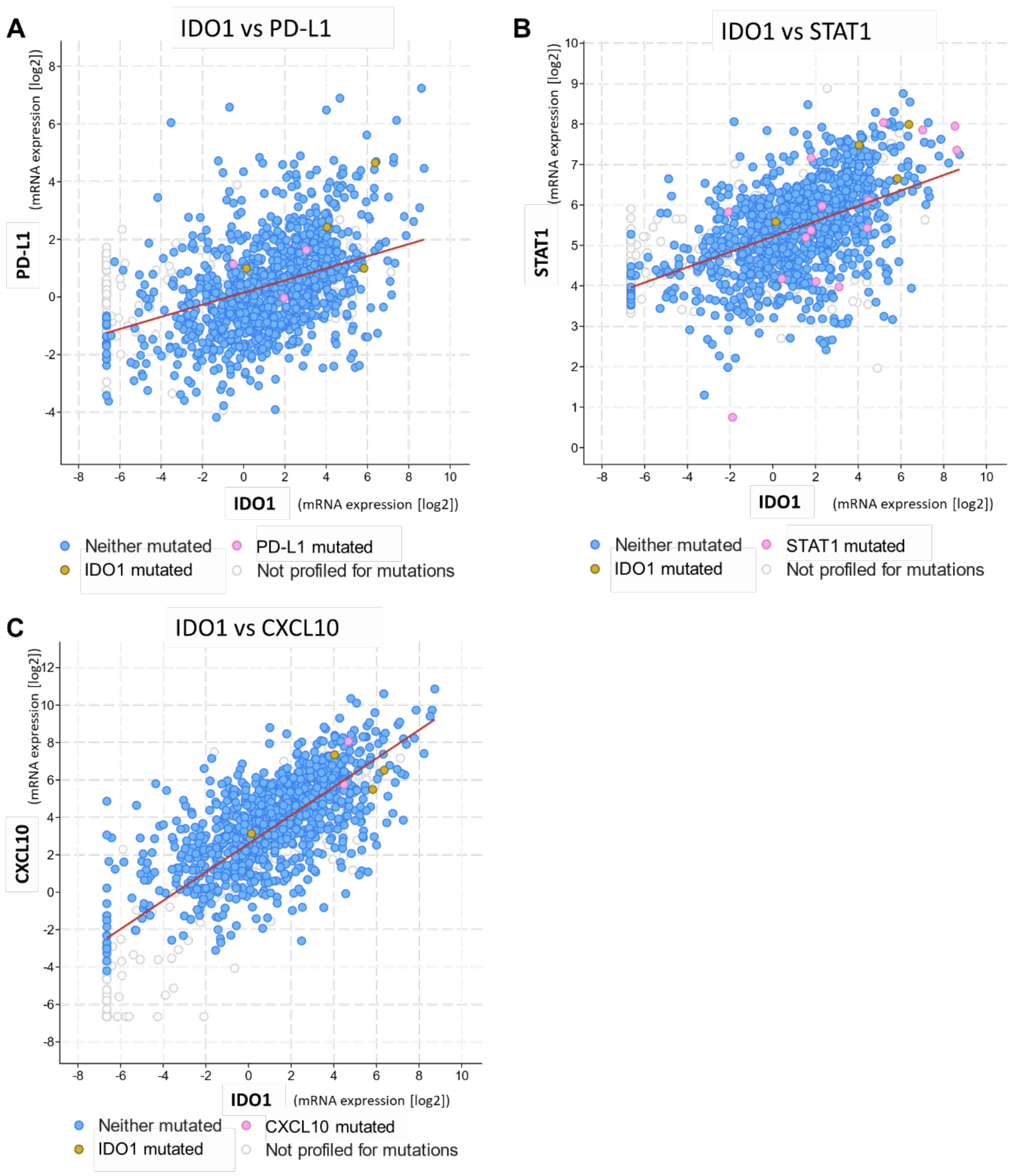

Correlation was tested using Spearman test with a p value threshold of 0.05 for significance. Correlation between (A) IDO1 vs. PD-L1, (B) IDO1 vs. STAT1, and (C) IDO1 vs. CXCL10 was compared by using TCGA data from 1210 samples that contained mRNA expression information to validate findings using our dataset. "mutated" in figure label means any type of alteration including mutation, amplification, and deletion. CXCL10 (Spearman R = 0.70, $p < 0.001$), STAT1 (Spearman R = 0.53, $p < 0.001$) and PD-L1 (Spearman R = 0.44, $p < 0.001$) were correlated with IDO1 expression. The data were accessed, and the figure was generated via cBioPortal for Cancer Genomics (https://www.cbioportal.org/) on 10/22/2023.

Figure S2. TCGA data analysis of mRNA expression of IDO1, AKT1, MTOR, and PIK3CA, related to Table 1 and Figure 3.

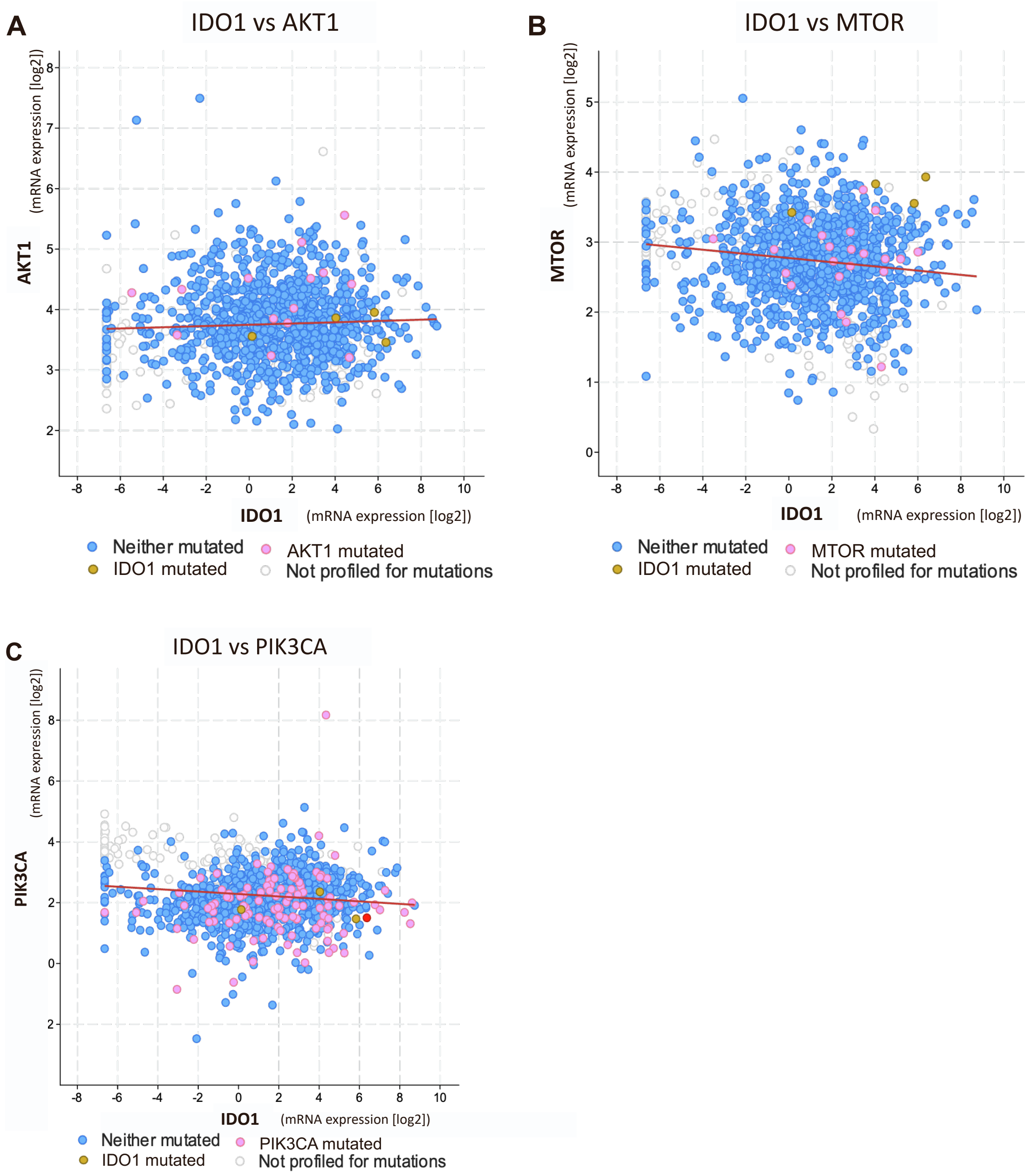

Correlation was tested using Spearman test with a p value threshold of 0.05 for significance. Correlation between (A) IDO1 vs. AKT1, (B) IDO1 vs. MTOR, and (C) IDO1 vs. PIK3CA was compared by using TCGA data from 1210 samples that contained mRNA expression information to validate findings using our dataset. “mutated” in figure label means any type of alteration including mutation, amplification, and deletion. MTOR was slightly correlated (Spearman R = -0.15, $p < 0.001$), but AKT1 (Spearman R = 0.03, p = 0.312) and PIK3CA (Spearman R = -0.04, p = 0.183) were not significantly correlated with IDO1 mRNA expression. The data were accessed, and the figure was generated via cBioPortal for Cancer Genomics (https://www.cbioportal.org/) on 10/22/2023.

Figure S3. Progression-free survival and overall survival based on PD-L1 and PD-1 expression in patients treated with immune checkpoint inhibitors in the first-line setting (n = 102), related to Table 2 and 3.

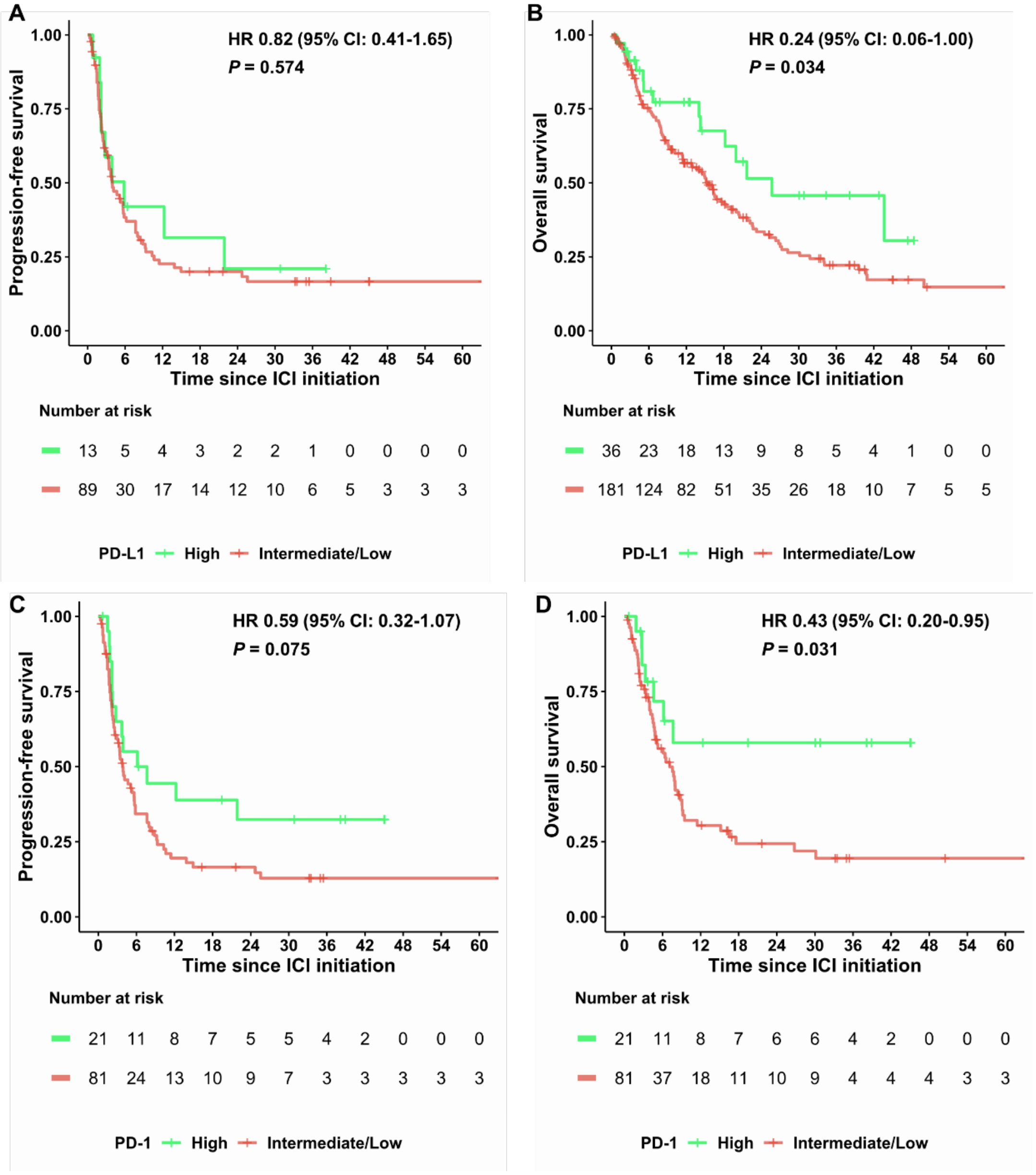

(**Panels A and B**) Progression-free survival and overall survival based on PD-L1 expression. (**Panels C and D**) Progression-free survival and overall survival based on PD-1 expression.

X axis: Time since initiation of immune checkpoint inhibitors. **Y axis:** PFS (A, C), OS (B, D).

Expression profiles were stratified by rank values into "low" (0-24), "intermediate" (25-74), and "high" (75-100).

Panels A and B: The Kaplan Meier curves according to PD-L1 high (n = 13) and intermediate/low (n = 89) group.

Panel A: PFS HR = 0.82 (95% CI: 0.41-1.65, p = 0.574)

Panel B: OS HR = 0.24 (95% CI: 0.06-1.00, p = 0.034).

Panels C and D: The Kaplan Meier curves according to PD-1 high (n = 21) and intermediate/low (n = 81) group.

Panel C: PFS HR = 0.59 (95% CI: 0.32-1.07, p = 0.075)

Panel D: OS HR = 0.43 (95% CI: 0.20-0.95, p = 0.031).

Abbreviations: 95% CI, 95% confidence interval; HR, hazard ratio; OS, overall survival; PD-1, Programmed cell death protein 1; PD-L1, Programmed death-ligand 1; PFS, progression-free survival.

Figure S4. Overall survival based on IDO1 and either PD-L1 or PD-1 expression in advanced cancer treated with immune checkpoint inhibitors in the first-line setting (N=102), related to Table 2.

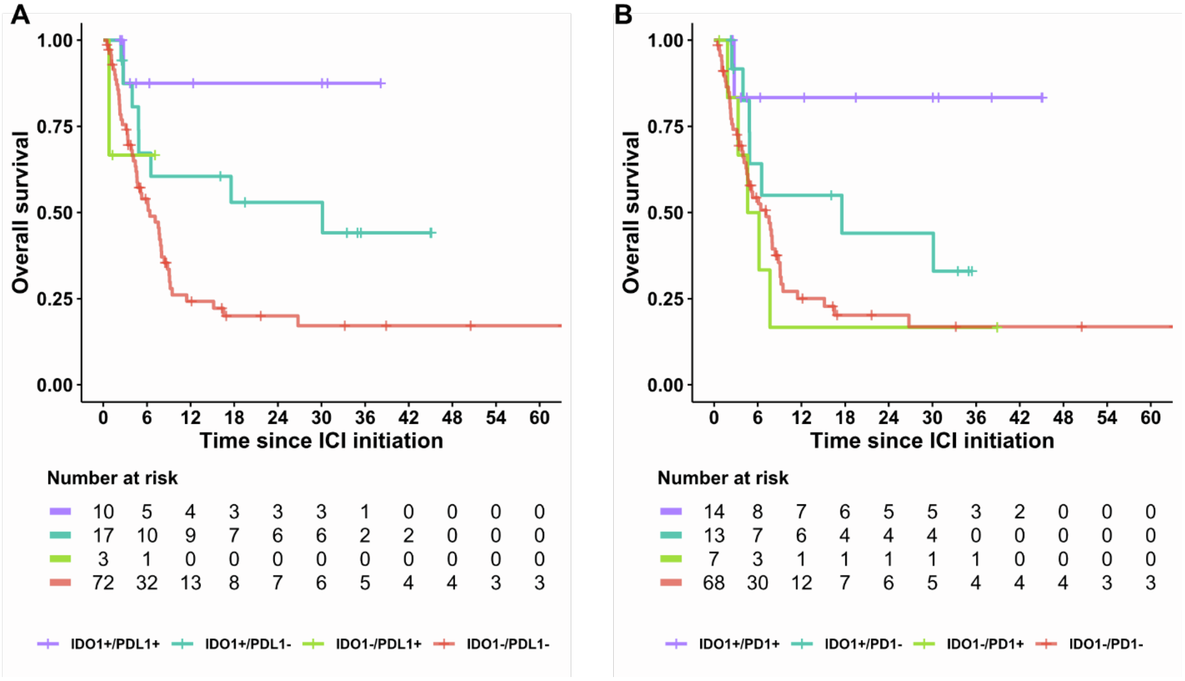

(**Panel A**) Kaplan-Meier curve based on IDO1 and PD-L1 expression, and (**Panel B**) IDO1 and PD-1 expression. X axis: Time since initiation of immune checkpoint inhibitors (months). Y axis: Probability of overall survival. Expression profiles were stratified by rank values into "not high" (0-74) and "high" (75-100) denoted as "+" and "-", respectively. "+" in figure labels mean the "high" group and "-" means the "intermediate/low" group.

Table S3. Summary table of overall survival results according to IDO1 and either PD-L1 or PD-1 expression in advanced cancer treated with immune checkpoint inhibitors in the first-line setting (N=102), related to Table 2. (**Panel A**) IDO1 and PD-L1, and (**Panel B**) IDO1 and PD-1 expression.

A	N of pt	Hazard ratio (95% CI)		
IDO1/PD-L1	102	Overall: p = 0.012*		
High/High	10	- (reference)	-	-
High/Not high	17	3.83 (0.48-30.7), p = 0.206	- (reference)	-
Not high/High	3	10.1 (0.62-163.0), p = 0.104	2.63 (0.32-21.4), p = 0.365	- (reference)
Not high/Not high	72	8.80 (1.21-63.8), p = 0.031*	2.30 (1.08-4.88), p = 0.031*	0.87 (0.12-6.37), p = 0.893

B	N of pt	Hazard ratio (95% CI)		
IDO1/PD-1	102	Overall: p = 0.008*		
High/High	14	- (reference)	-	-
High/Not high	13	3.81 (0.79-18.3), p = 0.095*	- (reference)	-
Not high/High	7	8.23 (1.59-42.6), p = 0.012*	2.16 (0.68-6.84), p = 0.190	- (reference)
Not high/Not high	68	7.01 (1.70-29.0), p = 0.007*	1.84 (0.83-4.09), p = 0.135	0.85 (0.34-2.14), p = 0.732

Figure S5. Progression-free survival based on IDO1 and either PD-L1 or PD-1 expression in advanced cancer treated with immune checkpoint inhibitors in the first-line setting (N=102), related to Table 3.

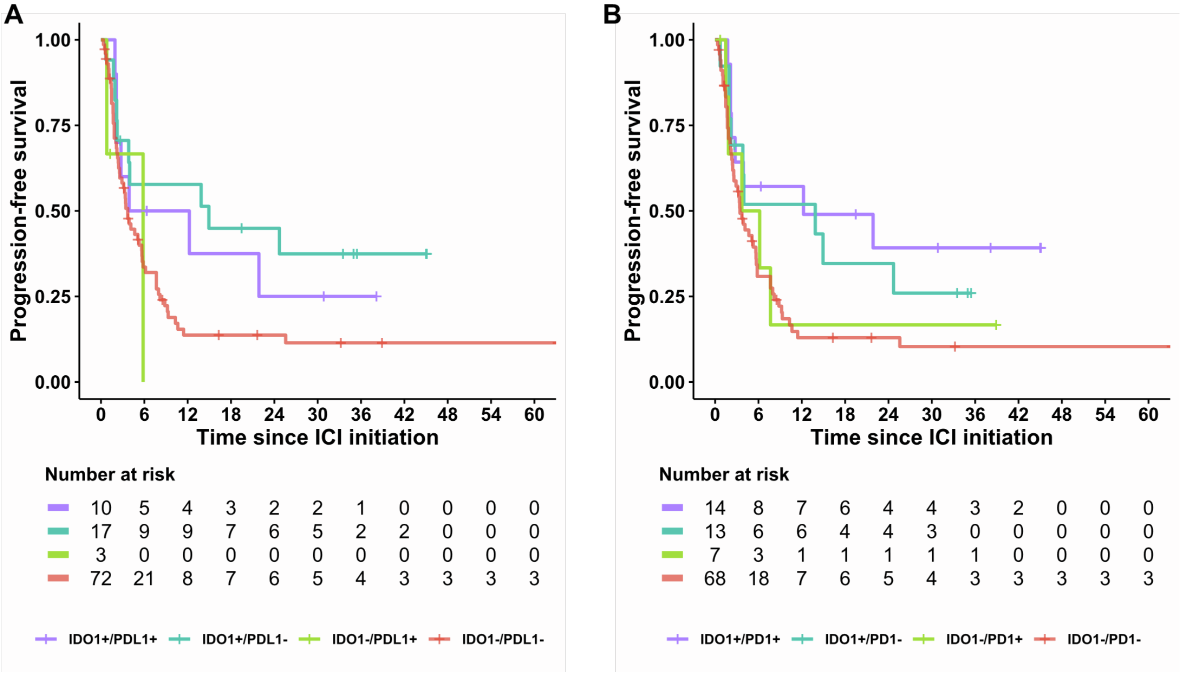

(**Panel A**) Kaplan-Meier curve based on IDO1 and PD-L1 expression, and (**Panel B**) IDO1 and PD-1 expression. X axis: Time since initiation of immune checkpoint inhibitors (months). Y axis: Probability of progression-free survival. Expression profiles were stratified by rank values into "not high" (0-74) and "high" (75-100) denoted as "+" and "-" respectively. "+" in figure labels mean the "high" group and "-" means the "intermediate/low" group.

Table S4. Summary table of progression-free survival results according to IDO1 and either PD-L1 or PD-1 expression in advanced cancer treated with immune checkpoint inhibitors in the first-line setting (N=102), related to Table 3. **(Panel A**) IDO1 and PD-L1, and (**Panel B**) IDO1 and PD-1 expression.

A	N of pt	Hazard ratio (95% CI)		
IDO1/PD-L1	102	Overall: p = 0.064		
High	10	- (reference)	-	-
High	17	0.75 (0.29-1.97), p = 0.560	- (reference)	-
Not high	3	2.62 (0.54-12.8), p = 0.232	3.50 (0.75-16.2), p = 0.110	- (reference)
Not high	72	1.68 (0.76-3.69), p = 0.197	2.24 (1.13-4.42), p = 0.020*	0.64 (0.16-2.64), p = 0.537

B	N of pt	Hazard ratio (95% CI)		
IDO1/PD-1	102	Overall: p = 0.072		
High	14	- (reference)	-	-
High	13	1.32 (0.51-3.42), p = 0.566	- (reference)	-
Not high	7	1.98 (0.65-6.08), p = 0.232	1.50 (0.50-4.49), p = 0.470	- (reference)
Not high	68	2.37 (1.12-5.00), p = 0.024*	1.79 (0.88-3.65), p = 0.110	1.19 (0.48-2.99), p = 0.705

Supplemental references. References for Resource S1, related to STAR Methods.

1. Chen L, Diao L, Yang Y, Yi X, Rodriguez BL, Li Y, *et al.* CD38-Mediated Immunosuppression as a Mechanism of Tumor Cell Escape from PD-1/PD-L1 Blockade. Cancer Discov **2018**;8:1156-75
2. Perrot I, Michaud HA, Giraudon-Paoli M, Augier S, Docquier A, Gros L, *et al.* Blocking Antibodies Targeting the CD39/CD73 Immunosuppressive Pathway Unleash Immune Responses in Combination Cancer Therapies. Cell Rep **2019**;27:2411-25.e9
3. Voron T, Colussi O, Marcheteau E, Pernot S, Nizard M, Pointet AL, *et al.* VEGF-A modulates expression of inhibitory checkpoints on CD8+ T cells in tumors. J Exp Med **2015**;212:139-48
4. Ohm JE, Gabrilovich DI, Sempowski GD, Kisseleva E, Parman KS, Nadaf S, *et al.* VEGF inhibits T-cell development and may contribute to tumor-induced immune suppression. Blood **2003**;101:4878-86
5. Aguilera TA, Giaccia AJ. Molecular Pathways: Oncologic Pathways and Their Role in T-cell Exclusion and Immune Evasion-A New Role for the AXL Receptor Tyrosine Kinase. Clin Cancer Res **2017**;23:2928-33
6. Gregory PA, Bracken CP, Smith E, Bert AG, Wright JA, Roslan S, *et al.* An autocrine TGF-beta/ZEB/miR-200 signaling network regulates establishment and maintenance of epithelial-mesenchymal transition. Mol Biol Cell **2011**;22:1686-98
7. Mariathasan S, Turley SJ, Nickles D, Castiglioni A, Yuen K, Wang Y, *et al.* TGF**β** attenuates tumour response to PD-L1 blockade by contributing to exclusion of T cells. Nature **2018**;554:544-8
8. Krummel MF, Allison JP. CD28 and CTLA-4 have opposing effects on the response of T cells to stimulation. J Exp Med **1995**;182:459-65
9. Jha V, Workman CJ, McGaha TL, Li L, Vas J, Vignali DA, *et al.* Lymphocyte Activation Gene-3 (LAG-3) negatively regulates environmentally-induced autoimmunity. PLoS One **2014**;9:e104484
10. Ascierto PA, Lipson EJ, Dummer R, Larkin J, Long GV, Sanborn RE, *et al.* Nivolumab and Relatlimab in Patients With Advanced Melanoma That Had Progressed on Anti-Programmed Death-1/Programmed Death Ligand 1 Therapy: Results From the Phase I/IIa RELATIVITY-020 Trial. J Clin Oncol **2023**;41:2724-35
11. Ishida Y, Agata Y, Shibahara K, Honjo T. Induced expression of PD-1, a novel member of the immunoglobulin gene superfamily, upon programmed cell death. Embo j **1992**;11:3887-95
12. Iwai Y, Ishida M, Tanaka Y, Okazaki T, Honjo T, Minato N. Involvement of PD-L1 on tumor cells in the escape from host immune system and tumor immunotherapy by PD-L1 blockade. Proc Natl Acad Sci U S A **2002**;99:12293-7
13. Latchman Y, Wood CR, Chernova T, Chaudhary D, Borde M, Chernova I, *et al.* PD-L2 is a second ligand for PD-1 and inhibits T cell activation. Nat Immunol **2001**;2:261-8
14. Johnston RJ, Comps-Agrar L, Hackney J, Yu X, Huseni M, Yang Y, *et al.* The immunoreceptor TIGIT regulates antitumor and antiviral CD8(+) T cell effector function. Cancer Cell **2014**;26:923-37
15. Fourcade J, Sun Z, Benallaoua M, Guillaume P, Luescher IF, Sander C, *et al.* Upregulation of Tim-3 and PD-1 expression is associated with tumor antigen-specific CD8+ T cell dysfunction in melanoma patients. J Exp Med **2010**;207:2175-86
16. Lines JL, Pantazi E, Mak J, Sempere LF, Wang L, O'Connell S, *et al.* VISTA is an immune checkpoint molecule for human T cells. Cancer Res **2014**;74:1924-32
17. Holmgaard RB, Zamarin D, Munn DH, Wolchok JD, Allison JP. Indoleamine 2,3-dioxygenase is a critical resistance mechanism in antitumor T cell immunotherapy targeting CTLA-4. J Exp Med **2013**;210:1389-402

18. Ino K, Yamamoto E, Shibata K, Kajiyama H, Yoshida N, Terauchi M, *et al.* Inverse correlation between tumoral indoleamine 2,3-dioxygenase expression and tumor-infiltrating lymphocytes in endometrial cancer: its association with disease progression and survival. Clin Cancer Res **2008**;14:2310-7
19. Witkiewicz AK, Costantino CL, Metz R, Muller AJ, Prendergast GC, Yeo CJ, *et al.* Genotyping and expression analysis of IDO2 in human pancreatic cancer: a novel, active target. J Am Coll Surg **2009**;208:781-7; discussion 7-9
20. Röhrig UF, Majjigapu SR, Caldelari D, Dilek N, Reichenbach P, Ascencao K, *et al.* 1,2,3-Triazoles as inhibitors of indoleamine 2,3-dioxygenase 2 (IDO2). Bioorg Med Chem Lett **2016**;26:4330-3
21. Fujiwara Y, Kato S, Nesline MK, Conroy JM, DePietro P, Pabla S, *et al.* Indoleamine 2,3-dioxygenase (IDO) inhibitors and cancer immunotherapy. Cancer Treat Rev **2022**;110:102461
22. Hsu YL, Hung JY, Chiang SY, Jian SF, Wu CY, Lin YS, *et al.* Lung cancer-derived galectin-1 contributes to cancer associated fibroblast-mediated cancer progression and immune suppression through TDO2/kynurenine axis. Oncotarget **2016**;7:27584-98
23. Platten M, Nollen EAA, Röhrig UF, Fallarino F, Opitz CA. Tryptophan metabolism as a common therapeutic target in cancer, neurodegeneration and beyond. Nat Rev Drug Discov **2019**;18:379-401
24. Litzenburger UM, Opitz CA, Sahm F, Rauschenbach KJ, Trump S, Winter M, *et al.* Constitutive IDO expression in human cancer is sustained by an autocrine signaling loop involving IL-6, STAT3 and the AHR. Oncotarget **2014**;5:1038-51
25. Kitamura T, Fujishita T, Loetscher P, Revesz L, Hashida H, Kizaka-Kondoh S, *et al.* Inactivation of chemokine (C-C motif) receptor 1 (CCR1) suppresses colon cancer liver metastasis by blocking accumulation of immature myeloid cells in a mouse model. Proc Natl Acad Sci U S A **2010**;107:13063-8
26. Flores-Toro JA, Luo D, Gopinath A, Sarkisian MR, Campbell JJ, Charo IF, *et al.* CCR2 inhibition reduces tumor myeloid cells and unmasks a checkpoint inhibitor effect to slow progression of resistant murine gliomas. Proc Natl Acad Sci U S A **2020**;117:1129-38
27. Cheng Y, Ma XL, Wei YQ, Wei XW. Potential roles and targeted therapy of the CXCLs/CXCR2 axis in cancer and inflammatory diseases. Biochim Biophys Acta Rev Cancer **2019**;1871:289-312
28. Hennequart M, Pilotte L, Cane S, Hoffmann D, Stroobant V, Plaen E, *et al.* Constitutive IDO1 Expression in Human Tumors Is Driven by Cyclooxygenase-2 and Mediates Intrinsic Immune Resistance. Cancer Immunol Res **2017**;5:695-709
29. Byrne KT, Vonderheide RH. CD40 Stimulation Obviates Innate Sensors and Drives T Cell Immunity in Cancer. Cell Rep **2016**;15:2719-32
30. Zappasodi R, Sirard C, Li Y, Budhu S, Abu-Akeel M, Liu C, *et al.* Rational design of anti-GITR-based combination immunotherapy. Nat Med **2019**;25:759-66
31. Soldevilla MM, Villanueva H, Meraviglia-Crivelli D, Menon AP, Ruiz M, Cebollero J, *et al.* ICOS Costimulation at the Tumor Site in Combination with CTLA-4 Blockade Therapy Elicits Strong Tumor Immunity. Mol Ther **2019**;27:1878-91
32. Curti BD, Kovacsovics-Bankowski M, Morris N, Walker E, Chisholm L, Floyd K, *et al.* OX40 is a potent immune-stimulating target in late-stage cancer patients. Cancer Res **2013**;73:7189-98
33. Sugamura K, Ishii N, Weinberg AD. Therapeutic targeting of the effector T-cell co-stimulatory molecule OX40. Nat Rev Immunol **2004**;4:420-31
34. Zhu Y, Knolhoff BL, Meyer MA, Nywening TM, West BL, Luo J, *et al.* CSF1/CSF1R blockade reprograms tumor-infiltrating macrophages and improves response to T-cell checkpoint immunotherapy in pancreatic cancer models. Cancer Res **2014**;74:5057-69

35. Mok S, Koya RC, Tsui C, Xu J, Robert L, Wu L, *et al.* Inhibition of CSF-1 receptor improves the antitumor efficacy of adoptive cell transfer immunotherapy. Cancer Res **2014**;74:153-61
36. Burger JA, Kipps TJ. CXCR4: a key receptor in the crosstalk between tumor cells and their microenvironment. Blood **2006**;107:1761-7
37. Hughes R, Qian BZ, Rowan C, Muthana M, Keklikoglou I, Olson OC, *et al.* Perivascular M2 Macrophages Stimulate Tumor Relapse after Chemotherapy. Cancer Res **2015**;75:3479-91
38. Tumeh PC, Harview CL, Yearley JH, Shintaku IP, Taylor EJ, Robert L, *et al.* PD-1 blockade induces responses by inhibiting adaptive immune resistance. Nature **2014**;515:568-71
39. Litchfield K, Reading JL, Puttick C, Thakkar K, Abbosh C, Bentham R, *et al.* Meta-analysis of tumor- and T cell-intrinsic mechanisms of sensitization to checkpoint inhibition. Cell **2021**;184:596-614.e14
40. Marcovecchio PM, Thomas G, Salek-Ardakani S. CXCL9-expressing tumor-associated macrophages: new players in the fight against cancer. J Immunother Cancer **2021**;9
41. Reschke R, Yu J, Flood B, Higgs EF, Hatogai K, Gajewski TF. Immune cell and tumor cell-derived CXCL10 is indicative of immunotherapy response in metastatic melanoma. J Immunother Cancer **2021**;9
42. Doi T, Muro K, Ishii H, Kato T, Tsushima T, Takenoyama M, *et al.* A Phase I Study of the Anti-CC Chemokine Receptor 4 Antibody, Mogamulizumab, in Combination with Nivolumab in Patients with Advanced or Metastatic Solid Tumors. Clin Cancer Res **2019**;25:6614-22
43. de Oliveira CE, Gasparoto TH, Pinheiro CR, Amôr NG, Nogueira MRS, Kaneno R, *et al.* CCR5-Dependent Homing of T Regulatory Cells to the Tumor Microenvironment Contributes to Skin Squamous Cell Carcinoma Development. Mol Cancer Ther **2017**;16:2871-80
44. Rudensky AY. Regulatory T cells and Foxp3. Immunol Rev **2011**;241:260-8
